# Mapping the molecular signature of ABA-regulated gene expression in germinating barley embryos

**DOI:** 10.1186/s12870-025-06654-z

**Published:** 2025-05-10

**Authors:** Ewa Sybilska, Bahareh Sadat Haddadi, Luis A. J. Mur, Manfred Beckmann, Szymon Hryhorowicz, Joanna Suszynska-Zajczyk, Monika Knaur, Andrzej Pławski, Agata Daszkowska-Golec

**Affiliations:** 1 Institute of Biology, Biotechnology and Environmental Protection, Faculty of Natural Sciences, University of Silesia in Katowice, Katowice, Poland; 2 Department of Life Science, Aberystwyth University, Aberystwyth, UK; 3 Institute of Human Genetics, Polish Academy of Sciences, Poznan, Poland; 4 Department of Biochemistry and Biotechnology, Poznan University of Life Sciences, Poznan, Poland; 5 Department of General, Endocrinological Surgery and Gastroenterological Oncology, Poznan University of Medical Sciences, Poznan, Poland

**Keywords:** ABA, Barley, Embryo, Metabolomics, Seed germination, Spatial transcriptomics, Transcriptomics

## Abstract

**Background:**

Abscisic acid (ABA) regulates key plant processes, including seed germination, dormancy, and abiotic stress responses. While its physiological role in germination is well-documented, the molecular mechanisms are still poorly understood. To address this, we analyzed transcriptomic and metabolomic changes in ABA-treated germinating barley (*Hordeum vulgare*) embryos. To map ABA-responsive gene expression across embryonic tissues, we employed the Visium Spatial Transcriptomics (10× Genomics). This approach, which remains technically challenging to be applied in plant tissues, enabled the precise localization of gene expression across six embryo regions, offering insights into tissue-specific expression patterns that cannot be resolved by traditional RNA-seq.

**Results:**

Transcriptomic analysis indicated that ABA acts primarily as a germination repressor. Gene ontology (GO) and the Kyoto Encyclopedia of Genes and Genomes (KEGG) enrichment analyses linked ABA-inhibited genes to energy metabolism, lignin biosynthesis, cell wall organization, and photosynthesis, while induced genes were associated with environmental adaptation and phytohormone signaling. Differentially expressed genes (DEGs) correlated with metabolites involved in phytohormone pathways, including gibberellins, jasmonates, brassinosteroids, salicylic acid, auxins, and ABA metabolism. Comparisons with developing seed transcriptomes suggested an ABA-associated gene expression signature in embryos. Spatial transcriptomics technique made possible the precise identification of ABA-induced transcriptional changes within distinct embryonic tissues.

**Conclusions:**

Integrating transcriptomics, metabolomics and spatial transcriptomics defined the molecular signature of ABA-induced modulation of phytohormonal crosstalk, energy metabolism, and tissue-specific gene activity in germinating seeds. The successful use of spatial transcriptomics adds a novel layer of resolution for understanding tissue-specific ABA responses during barley seed germination. These findings offer new insights into the ABA role in seed germination and potential strategies for enhancing crop resilience.

**Supplementary Information:**

The online version contains supplementary material available at 10.1186/s12870-025-06654-z.

## Background

Abscisic acid (ABA) is a key regulator of seed dormancy, preventing premature germination under unfavorable conditions [1, 2]. In order to maintain dormancy, ABA synthesized *de novo* in the embryo plays a crucial role, but ABA produced in maternal tissues only plays a contributory role [3, 4]. The suppressive action of ABA is closely linked to its agonistic role with gibberellins (GA). ABA inhibits the expression of GA biosynthetic genes whilst strengthening the endosperm cell wall to delay germination [5]. In Arabidopsis, mutants defective in ABA biosynthesis or signaling exhibit germination rate, whereas GA biosynthesis mutants do not germinate in the absence of exogenous GA [6–10]. The balance between ABA and GA is controlled by a complex transcriptional network. ABSCISIC ACID INSENSITIVE 3 (ABI3) and ABI5 transcription factors regulate the expression of the *MOTHER OF FT AND TFL1* (*MFT*) genes through a negative feedback loop mechanism in the ABA signaling pathway [11]. Importantly, crosstalk between ABA and other phytohormones is also crucial for controlling seed germination and dormancy. Auxins increase ABA levels and inhibit GA synthesis, which delays germination [12, 13]. However, the effect of auxins on seed germination appears to be dose-dependent. High auxin concentrations promote dormancy, whereas low auxin concentrations promote germination [14]. Ethylene (ET) reduces ABA accumulation by both inhibiting its synthesis and promoting its inactivation, and by negatively regulating ABA signaling [15]. Brassinosteroids (BR) promote seed germination via an *MFT*-dependent pathway and regulate starch degradation in the endosperm by modulating α-amylase expression [16, 17]. BRASSINOSTEROID INSENSITIVE 2 (BIN2) kinase binds to the ABI5 protein and phosphorylates it to influence ABA signaling [18]. Salicylic acid (SA) has been shown to inhibit germination under normal conditions, whereas it supports germination under salt stress by reducing oxidative damage [19]. SA also inhibits germination in a dose-dependent manner [20]. Cytokinins (CTK) act antagonistically to ABA, reducing *ABI5* expression and promoting germination [21]. The role of jasmonate (JA) is complex. The JA precursor, oxylipin 12-oxo-phytodienoic acid (OPDA), enhances ABA signaling, while jasmonoyl-l-isoleucine (JA-Ile) reduces dormancy [22, 23]. However, the effects of JA may be species-dependent. In wheat, JA stimulates seed germination; however, in Arabidopsis, jasmonate ZIM-domain (JAZ) proteins inhibit the activity of ABI3 and ABI5 to reduce the ABA signal [24, 25]. Other reports have suggested that JA in combination with auxins supports ABA function, leading to the inhibition of seed germination [26, 27].

Despite the increasing use of technologies integrating transcriptome and metabolome data in plant research, detailed analyses of the regulatory mechanisms underlying ABA responses during seed germination remain limited [28]. These types of studies have shown that ABA affects germination by regulating sugar metabolism and the cell wall in rapeseed, inhibiting photosynthesis and secondary metabolism in pear, and inducing seed dormancy via the *NCED6* gene in Arabidopsis [29–31]. Additionally, new information can be obtained through such technologies as Visium Spatial Transcriptomics (10× Genomics), which allows for high-resolution mapping of gene expression in specific plant tissues. A recent study by Peirats-Llobet et al. (2023) demonstrated the potential of spatial transcriptomics in plant research, focusing on germinating seeds [32]. This study provides a detailed spatial map of gene expression during seed germination, uncovering key regulatory networks and tissue-specific transcriptional activities that govern this critical developmental process.

In this study, we integrated transcriptomic and metabolomic analyses to elucidate the effects of ABA on barley embryo germination. This multi-omics strategy revealed coordinated interactions between ABA and other phytohormones, pinpointing the key genes and metabolites involved in this crosstalk. By comparing the transcriptomes of ABA-treated embryos and developing seeds, we delineated a common ABA-responsive gene set and identified genes uniquely regulated during germination. Furthermore, spatial transcriptomics enabled us to surpass the limitations of bulk RNA-seq by precisely localizing ABA-influenced gene expression across distinct embryo tissues. Together, these provided the molecular signature of ABA effects during seed germination.

## Methods

### Plant material and ABA treatment conditions

In our study, we used the spring barley cultivar ‘Sebastian’ that was selected due to its high yield potential, good malting quality, resistance to lodging, and strong resistance to stem rust (*Puccinia graminis*) and leaf rust (*Puccinia hordei*). Previously, we used ‘Sebastian’ as a parent variety to create the *Hor*TILLUS population [33]. The initial seeds were obtained from HODOWLA ROŚLIN STRZELCE Sp. z o.o IHAR Group, Poland. Subsequently, seeds were multiplied in our laboratory and collected from plants grown in the greenhouse of the Institute of Biology and Biotechnology in Katowice, Poland.

Barley (*Hordeum vulgare*) embryos of the ‘Sebastian’ cultivar were isolated from germinating seeds in the presence of 75 µM abscisic acid (ABA) and under control conditions at 1 day after imbibition (DAI). This concentration of ABA had been previously optimized based on dose–response experiments, as it enables differentiation between ABA-sensitive and ABA-insensitive genotypes, as shown in our previous study [34]. The ABA treatment started from sterilized seeds and continued until one day after imbibition (1 DAI) up to the moment of embryo isolation. Firstly, the barley seeds were surface-sterilized in a 20% sodium hypochlorite solution for 20 min, and rinsed thoroughly three times in sterile distilled water for 5 min per wash. Subsequently, the seeds were placed in 90 mm Petri dishes lined with three layers of Whatman filter paper and moistened with 5 ml of either sterile distilled water (control) or distilled water supplemented with 75 µM ABA (cis–trans-abscisic acid; Sigma-Aldrich, cat. 862169; Sigma-Aldrich). The seeds were stratified at 4 °C in the dark for four days to synchronize germination. After stratification, the Petri dishes were transferred to a growth chamber set to 22 °C, with a photoperiod of 16 h light / 8 h dark and a light intensity of 200 µmol m⁻² s⁻¹. Embryos were collected at 1 DAI and preserved in RNAlater™ Stabilization Solution (Thermo Fisher Scientific, cat. AM7020) until RNA isolation.

### RNA extraction, cDNA library construction and sequencing

RNA was extracted from four biological replicates, each consisting of 20 ‘Sebastian’ embryos isolated at 1 DAI under control conditions or in the presence of 75 µM ABA. Total RNA from each sample was isolated according to the manufacturer’s instructions using the mirVana™ Isolation Kit (Ambion, USA). RNA concentration and quality were assessed using a NanoDrop spectrophotometer and Agilent Bioanalyzer (Agilent Technologies, Santa Clara, CA, USA). The RNA library was constructed using the TruSeq stranded mRNA cDNA library preparation technique, followed by next-generation sequencing (NGS) at Macrogen Inc., South Korea. Sequencing was performed using an Illumina NovaSeq6000 system (40 million paired-end reads with a length of 150 bp). The initial quality assessment of the raw reads was performed using FastQC, and adapters were trimmed using the Cutadapt tool [35]. Quality control was re-evaluated after trimming. Poor-quality reads were removed using the Cutadapt software [35]. The cleaned paired-end reads were then aligned to the barley reference transcriptome BaRTv2.18 using Kallisto software [36, 37]. The mapped reads were quantified and normalized to transcripts per million (TPM) with Kallisto [36].

### Identification of differentially expressed genes

Differential expression analysis was performed using the limma-voom pipeline in the 3D-RNA-seq platform [38]. The comparison was made between ‘Sebastian’ embryos under control conditions and those treated with ABA (ABA.WT vs. control.WT). Differentially expressed genes (DEGs) were identified based on a significance threshold of log2FC ≥ 1.5 or ≤ -1.5, with a p-value < 0.01, adjusted using the Benjamini-Hochberg method.

### Gene function annotation

Gene Ontology (GO) enrichment analysis of differentially expressed genes (DEGs) was performed using the TopGO package (version 2.50.0) in R (version 4.2.1) with an adjusted p-value threshold of < 0.01 [39]. The results were visualized in RStudio using the ggplot2 package (version 3.5.1) [40] (https://rstudio.com/). For the Kyoto Encyclopedia of Genes and Genomes (KEGG) enrichment analysis, enriched pathways were identified using the clusterProfiler package (version 4.10.1) in RStudio, with a corrected p-value cutoff criterion of < 0.01 [41]. The KO identifiers of the DEGs used as input were obtained using the BlastKOALA tool (https://www.kegg.jp/blastkoala/) by querying the KEGG ORTHOLOGY (KO) database. Plots were generated in RStudio using the ggplot2 package (version 3.5.1) [40] (https://rstudio.com/).

### Prediction of transcription factors (TFs) and their binding sites

Transcription factors (TFs) were predicted using the PlantRegMap tool (https://planttfdb.gao-lab.org/prediction.php). Promoter sequences, corresponding to the 1500 bp regions upstream of the target genes, were extracted using the BioMart tool in EnsemblPlants v. 45, utilizing the MorexV3 barley genome version available in the EnsemblPlants database (http://plants.ensembl.org/biomart/martview/). Potential transcription factor-binding sites (TFBS) were targeted using the PlantRegMap Binding Site Prediction feature (https://plantregmap.gao-lab.org/binding_site_prediction.php). The resulting datasets of TFs and their associated target genes were integrated to assess the possible regulatory pairs.

### Metabolome analysis

The metabolomic has been previously described by Sybilska et al. (2024) [34]. Embryos from germinating seeds were ground into fine powder using liquid nitrogen and then chilled on ice. Then, 40 ± 1 mg of tissue was transferred into microcentrifuge tubes, followed by the addition of 1 mL of chloroform: methanol: ddH_2_O mixture (1:2.5:1 v/v). The samples were thoroughly mixed by vortexing at 4 °C for 15 min, and then returned to ice. Subsequently, they were centrifuged at 5000×g for 3 min at 4 °C. The resulting supernatants containing polar and nonpolar metabolites were carefully collected in fresh tubes and dried at 25 °C using a Buchi Rotavapor system to prevent complete evaporation. A final volume of 100 µL was retained for metabolomic analysis via liquid chromatography-tandem mass spectrometry (LC-MS/MS), as previously described by Baptista et al. (2018) [42]. Partial Least Squares Discriminant Analysis (PLS-DA) was used with the holomics R package [43].

### Integrative transcriptomic and metabolomic analysis

Integrative omic assessments of transcriptomic data were undertaken using the holomics R package in RStudio (version 2023.12.0) [43] (https://rstudio.com/).

### Comparative transcriptome data assessments

Transcriptomic profiles generated in this study for germinating ‘Sebastian’ embryos were compared with the developmental expression in barley seeds described by Kovacik et al. (2024) [44]. Differentially expressed genes (DEGs) from our study and their BaRTv2.18 gene IDs were translated into their corresponding HORVU.MOREX identifiers. The BaRTv2.18 dataset is the most recent barley reference transcriptome, based on the Barke cultivar, whereas the HORVU.MOREX identifiers correspond to the older Morex reference genome [37]. Due to differences in reference datasets, in a small number of cases, BaRTv2.18 identifiers were mapped to the same HORVU.MOREX identifier. Thus, 3,621 DEGs (65%) were mapped to the corresponding HORVU.MOREX identifiers and used for cross-study analysis. Splice variants of the same gene were counted as a single DEG.

### Spatial gene expression analysis in germinating barley embryos

#### Preparation of barley embryo sections

From isolated (cv. Sebastian) embryos germinated under control conditions or with 75 µM ABA at 1 day after imbibition (DAI), the embryonic root was removed. Next, the embryos were placed in an optimal cutting temperature (OCT) medium and then frozen in an isopentane bath on dry ice. Frozen embryos were stored at − 80 °C, and then cut into 10 μm thick sections in a cryostat (Leica CM3050 S) at − 18 °C. The embryo sections were placed on the Visium Spatial Gene Expression Slide. Embryo RNA was isolated using the RNeasy Mini Kit (Qiagen, Hilden, Germany). The RNA Integrity Number (RIN) was evaluated to determine the degree of RNA degradation using the Agilent 2100 Bioanalyzer. Slides were fixed in chilled methanol for 30 min at − 20 °C. After fixation, sections were stained for 5 min with 0.1% Safranin O (Sigma-Aldrich, cat. S8884-25G) in 50% ethanol. The sections were then washed in an alcohol series (50%, 70%, 100%) for 1 min. Slides were imaged in the bright field using a light microscope (Leica DS5500).

#### Tissue optimization (TO) procedure

To pre-permeabilize the tissue, the slides were assembled in a Visium slide cassette and incubated in pre-permeabilization solution (48 µl 10x Exonuclease I buffer (ThermoScientific, #EN0581); 4.5 µl of Bovine Serum Albumin (BSA), 10% Aqueous Solution, nuclease-free, Sigma-Aldrich, cat. 126615-25 ml,; and 2% (w/v) polyvinylpyrrolidone PVP40, Sigma-Aldrich, cat no. PVP40-500 g) at 37 °C for 30 min. This was followed by washing in 100 µl 0.1 × saline-sodium citrate (SSC) buffer (Sigma-Aldrich, cat. S6639L). The sections were permeabilized with Permeabilization mix™ (10x Genomics) at 37 °C for different times (2, 4, 6, 12, 18, and 24 min Tissue Optimization (TO) slides, including positive and negative control) or 6 min (Gene Expression (GE) slides). The wells were washed with 100 µl of 0.1× SSC buffer. After permeabilization, reverse transcription mixture™ (10x Genomics) was added to each section and incubated at 53 °C for 45 min, as described in the 10x Genomics User Guide (PN-1000186, CG000239_VisiumSpatialGeneExpression_UserGuide_RevD).

#### Tissue removal and washes (TO slide only)

To remove the tissue, a hydrolytic enzyme mixture was prepared by adding 70 µl of cellulase (Yakult -‘ONOZUKA’ R-10, cat. YAKL0012), pectate lyase (cat. E-PCLYAN2), and xyloglucanase (Megazyme, cat. E-XEGP), endo 1,4 β-xylanase (Megazyme, cat. E-XYNACJ), endo 1,4 β-mannanase (Megazyme, cat. E-BMACJ), and lichenase (Megazyme, cat. E-LICHN) to 140 µl of 250 mM sodium citrate (Sigma-Aldrich, cat. S-4641–1 kg). The enzymatic mixture was added to the wells, individual reaction chambers within the Visium Slide Cassette, and incubated in an IKA Mixer at 37 °C for 90 min with shaking (300 rpm). The wells were washed with 100 µl 0.1× SSC buffer. Samples were incubated with 10% H_2_O Triton X-100 solution (Sigma-Aldrich, cat. 93443-500 ml) in an IKA Mixer at 56 °C for 1 h with shaking (300 rpm), followed by a wash with 0.1× SSC buffer. Next wash consisted of a mixture of RLT buffer (Qiagen ref.79216) with 1% (v/v) β-mercaptoethanol, which was incubated in a Thermo Mixer at 56 °C for 1 h with shaking (300 rpm), followed by a wash with 0.1× SSC buffer. A final incubation with 70 µl proteinase K mixture (60 µl of proteinase K (Qiagen, cat. 19131), and 420 µl of PKD buffer (Qiagen, cat no. 1034963) was performed in a Thermo Mixer at 56 °C for 1 h with shaking (300 rpm). Hybridization chamber was detached, and the slide was washed in a Petri dish with 50 °C pre-warmed wash buffer 1 (2× SSC/0.1% sodium dodecyl sulfate (SDS) at 50 °C for 10 min with shaking (300 rpm). The slides were further washed with wash buffer 2 (0.2× SSC) and wash buffer 3 (0.1× SSC) at RT for 1 min with shaking (300 rpm). The slide was spin-dried in a swing-bucket centrifuge.

The tissue GE slide was then processed according to the Visium Spatial Gene Expression User Guide protocol.

### cDNA sequencing and differential gene expression analysis

cDNA sequencing was performed on a NovaSeq 6000 platform (Illumina) in paired-end mode with a read length of 151 bp (Macrogen, The Netherlands). Read sequence analysis was performed with Space Ranger v3.1.0 using the barley reference genome, cv. MorexV3 [45] (https://www.10xgenomics.com/support/software/space-ranger/latest). Data visualization results were processed using Loupe Browser 8 (https://www.10xgenomics.com/support/software/loupe-browser/). Differential gene expression (DEG) analysis between the ABA-treated and control samples was performed in six clusters within the germinating embryo: coleoptile, cotyledon, mesocotyl, plumule, scutellum, and radicle. Genes with low average abundance were excluded, and only DEGs with p-value ≤ 0.05 and log2FC ≥ 0.25 were included in further analysis.

### Statistical analyses

Statistical analyses, including calculation of correlation coefficients and generation of plots, were performed using RStudio (version 2023.12.0) (https://rstudio.com/).

## Results

### ABA suppresses the expression of numerous genes in germinating embryos

**Fig. 1:**
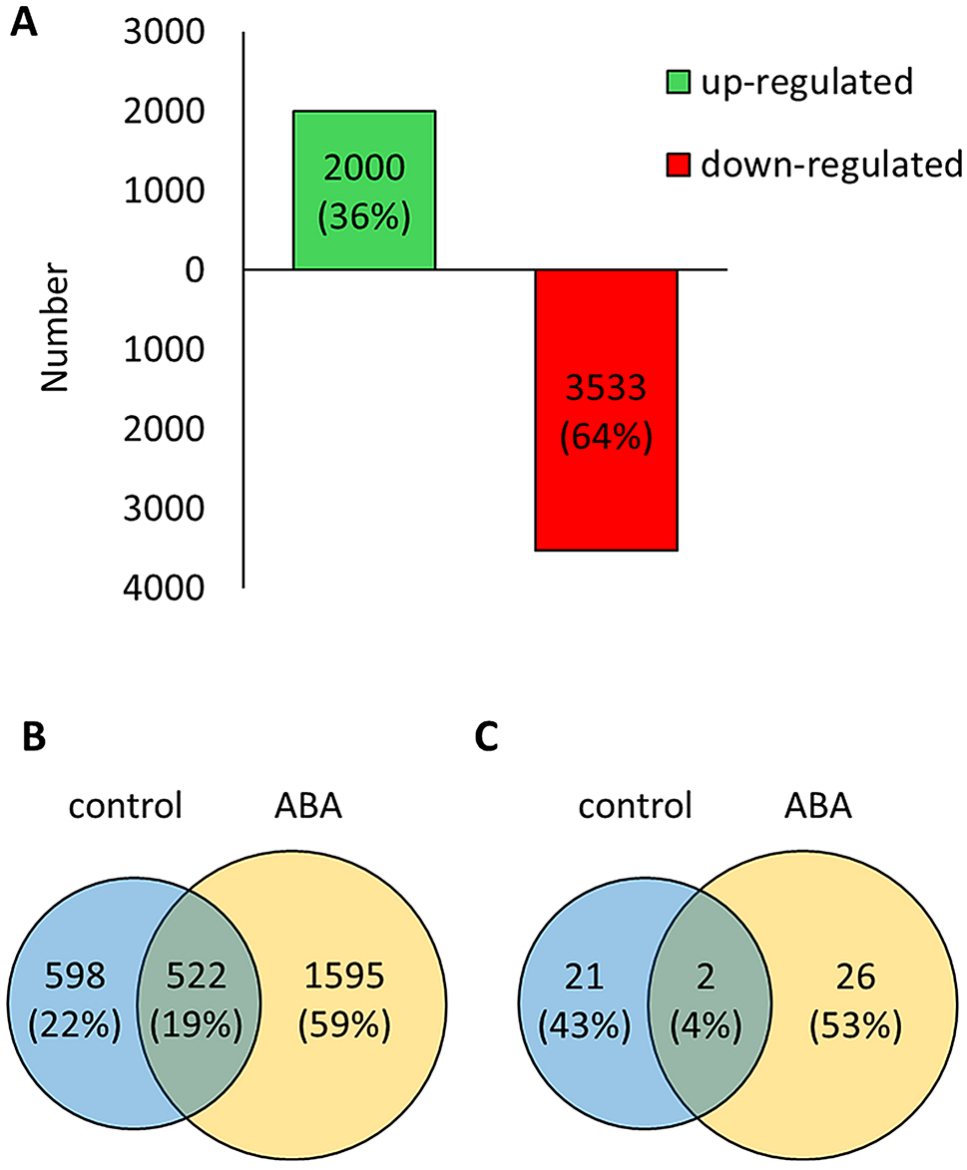
Differentially expressed genes (DEGs) in barley embryos germinating in the presence of 75 µM ABA compared to control conditions. (**A**) Number of upregulated and downregulated DEGs. (**B**) Venn diagram of poorly expressed genes (0–1 transcript per million [TPM]). (**C**) Venn diagram displaying the highly expressed genes (> 1000 TPM)

To identify genes regulated by ABA during early seed germination, we analyzed transcriptomic changes in germinating embryos of the barley variety ‘Sebastian’ in the presence of 75 µM ABA versus control conditions at 1 DAI (Additional file 1: Data S1). A total of 5,533 differentially expressed genes (DEGs) were detected of which 3,533 (64%) were downregulated, while 2,000 (36%) were upregulated (Fig. 1A). Of the total, 2,715 DEGs exhibited low expression levels (TPM ≤ 1) under both control and ABA treatments but 1,595 genes (59%) showed reduced levels with ABA (Fig. 1B). Only 49 DEGs displayed high expression (TPM > 1000) in either treatment (Fig. 1C). Hence, ABA could be acting as repressor of gene expression during seed germination.

### Transcriptional regulation of ABA-treated germinating embryos

**Fig. 2:**
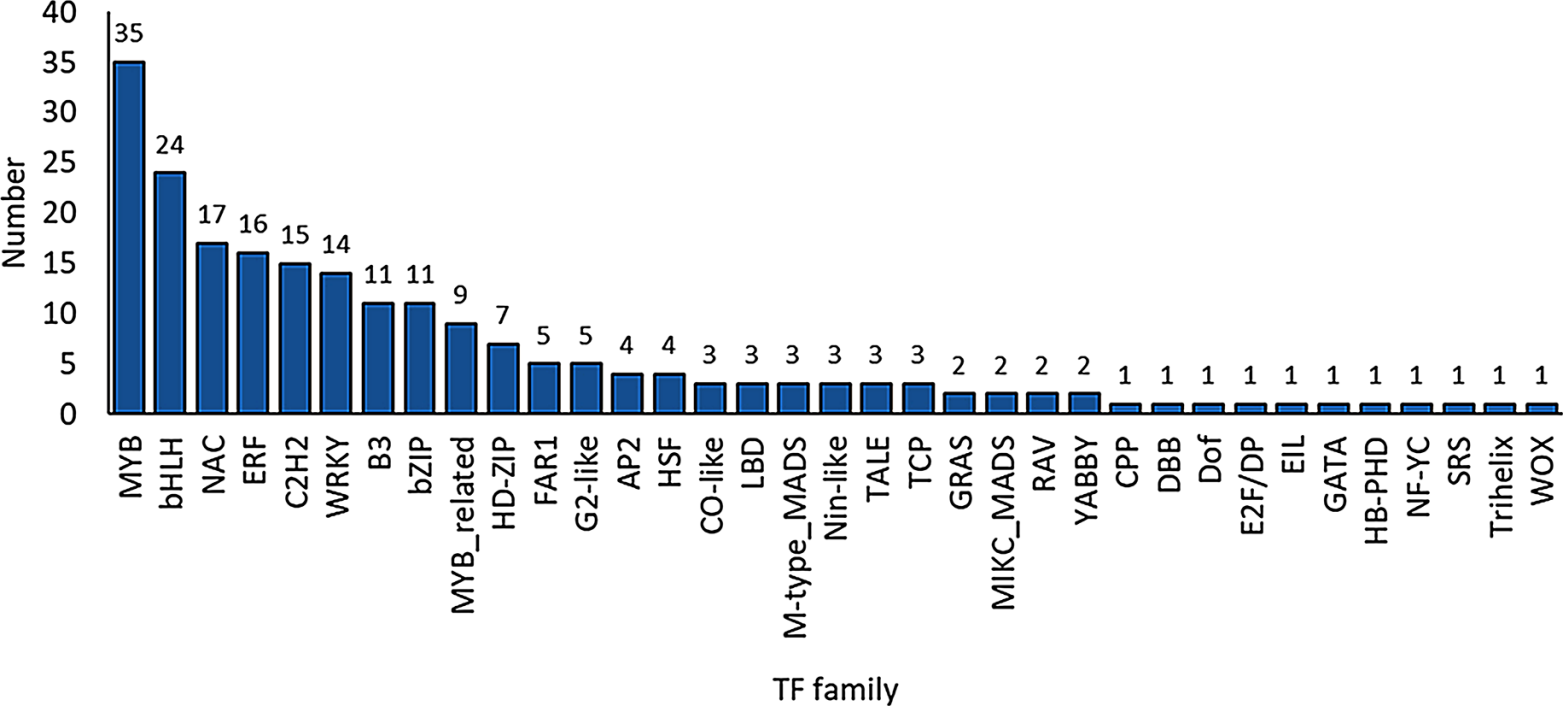
Transcription factors (TFs) in barley embryos germinating in the presence of 75 µM ABA compared to control conditions

Given ABA effects on gene expression during germination, we focused on identifying the regulatory transcription factors. A total of 214 transcription factors (TFs) were identified. These belonged to 35 TF families, with the MYB family the most highly represented (35 genes) (Fig. 2; Additional file 2: Data S2). To determine whether the identified TFs could potentially regulate the expression of DEGs, we screened for binding sites within DEG promoter sequences. Of the 214 TFs, 23 had binding sites within 3,617 DEGs (Table 1; Additional file 3: Data S3). Several TFs specifically associated with the abscisic acid-activated signaling pathway (GO:0009738), including homologs of crucial ABA regulators such as AtABI3 (ABSCISIC ACID INSENSITIVE 3, BaRT2v18chr3HG161790), AtAREB3 (ABA-RESPONSIVE ELEMENT BINDING PROTEIN 3, BaRT2v18chr1HG033690), AtABF3 (ABSCISIC ACID-RESPONSIVE ELEMENT-BINDING FACTOR 3, BaRT2v18chr3HG156370). ABI3 acts as the main regulator that controls seed dormancy and activates the ABA response. AREB3 and ABF3 further enhance the action of ABA by binding to ABRE (ABA-responsive elements) in target gene promoters, thereby intensifying the inhibitory effect on germination and effectively maintaining seeds in a dormant state [46–49]. This suggests a substantial regulatory influence of a limited number of TFs on the transcriptional response to ABA during seed germination.

**Table 1:** Identified transcription factors (TF) with binding sites within DEGs

BaRTv2 ID	TF family	Gene annotation	log2FC	Number of target genes	Gene Arabidopsis	Arabidopsis gene name
BaRT2v18chr2HG106120	ERF	Ethylene-responsive transcription factor	3.91	2271	AT5G25190	ESE3
BaRT2v18chr1HG028050	ERF	AP2/ERF domain-containing protein	2.07	1900	AT2G47520	ERF71
BaRT2v18chr3HG146510	B3	TF-B3 domain-containing protein	2.67	1888	AT1G28300	LEC2
BaRT2v18chr4HG208310	C2H2	C2H2-type domain-containing protein	2.78	1609	AT3G50700	IDD2
BaRT2v18chr1HG030570	MIKC_MADS	PISTILLATA-like MADS-box transcription factor	-2.13	1530	AT5G20240	PI
BaRT2v18chr3HG161790	B3	Transcription factor VP-1 homologue	2.80	1345	AT3G24650	ABI3
BaRT2v18chr1HG046320	bZIP	G-box binding factor	2.78	1067	AT4G01120	GBF2
BaRT2v18chr6HG319320	HD-ZIP	Homeobox-leucine zipper family protein	-1.90	1062	AT1G17920	HDG12
BaRT2v18chr5HG226660	bZIP	BZIP transcription factor family	-4.58	1038	AT3G54620	BZIP25
BaRT2v18chr3HG156370	bZIP	ABA response element binding factor	3.63	1021	AT4G34000	ABF3
BaRT2v18chr1HG033690	bZIP	BZIP transcription factor	1.75	991	AT3G56850	AREB3
BaRT2v18chr3HG119680	RAV	AP2/B3 transcription factor family protein	-3.57	964	AT3G25730	EDF3
BaRT2v18chr2HG077710	bHLH	BHLH domain-containing protein	2.06	931	AT3G59060	PIL6
BaRT2v18chr3HG147640	WRKY	WRKY transcription factor	-2.76	875	AT1G29280	WRKY65
BaRT2v18chr1HG011410	GATA	GATA-type domain-containing protein	-1.78	817	AT3G06740	GATA15
BaRT2v18chr2HG059890	G2-like	Two-component response regulator	2.11	790	AT3G25790	HHO1
BaRT2v18chr2HG092760	TCP	TCP transcription factor	-3.42	785	AT5G23280	TCP7
BaRT2v18chr2HG058120	NAC	NAC domain-containing protein	-1.91	765	AT5G61430	NAC100
BaRT2v18chr1HG014170	MYB	HTH myb-type domain-containing protein	-4.62	691	AT5G11510	MYB3R-4
BaRT2v18chr3HG150290	TCP	TCP family transcription factor containing protein	-1.99	584	AT5G60970	TCP5
BaRT2v18chr4HG205400	AP2	AP2 domain containing protein	1.95	578	AT4G37750	ANT
BaRT2v18chr1HG030650	NAC	NAC domain-containing protein	3.19	528	AT1G01720	ATAF1
BaRT2v18chr6HG317740	E2F/DP	E2F transcription factor	-2.38	418	AT3G01330	DEL3

### The role of ABA-regulated genes in germinating barley embryos

**Fig. 3:**
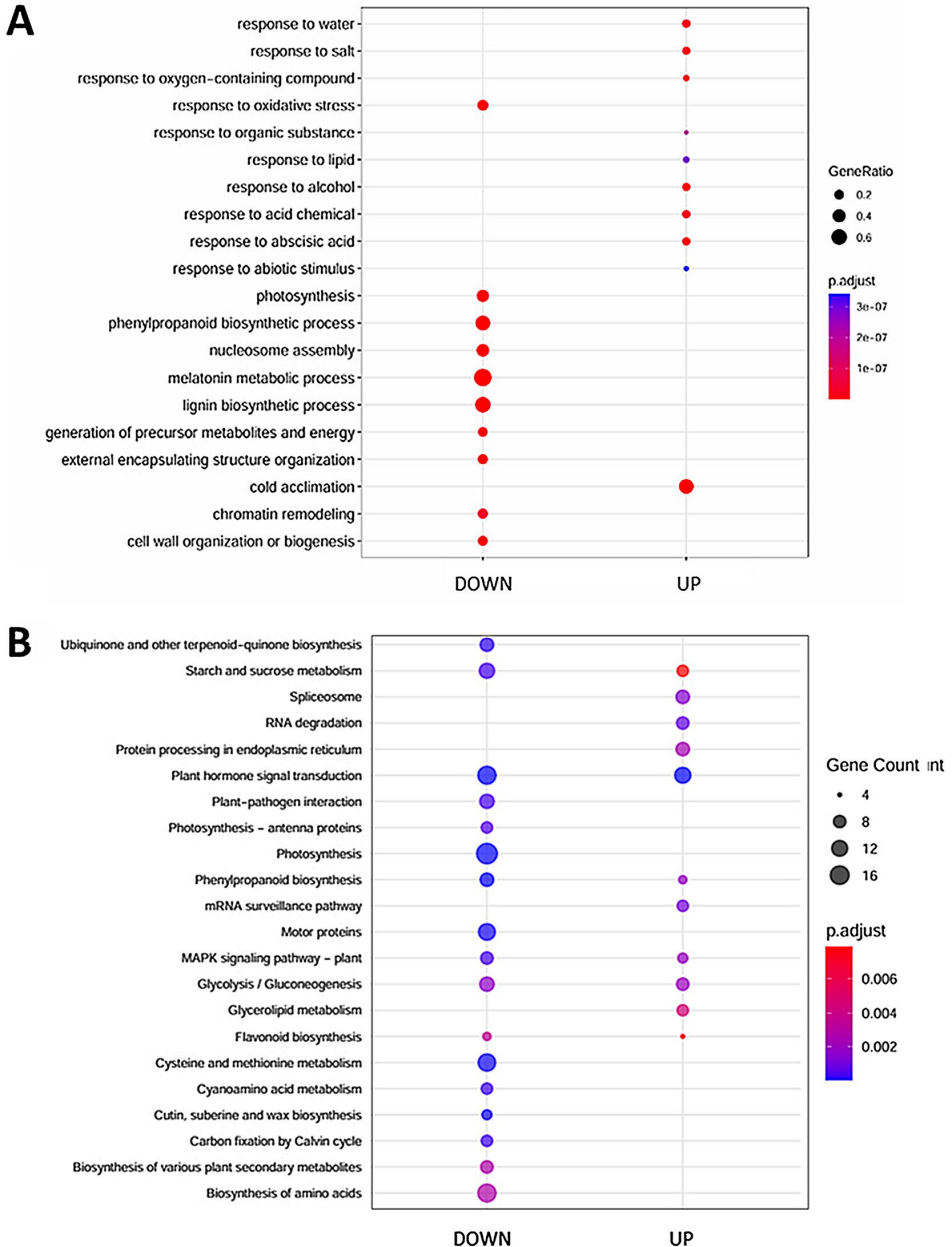
GO enrichment and KEGG analysis of up- and downregulated genes differentially expressed genes (DEGs) in barley embryos germinating in the presence of 75 µM ABA compared to control conditions. (**A**) GO analysis. (**B**) KEGG pathway analysis. GO (Gene Ontology), KEGG (Kyoto Encyclopedia of Genes and Genomes)

Gene Ontology (GO) analysis was used to highlight the biological processes associated with DEGs linked to ABA treatment (Fig. 3A; Additional file 4: Data S4; Additional file 5: Data S5). ABA downregulated genes were predominantly associated with processes such as cell wall organization or biogenesis (GO:0071554), phenylpropanoid biosynthetic process (GO:0009699), external encapsulating structure organization (GO:0045229), melatonin metabolism (GO:0030186), photosynthesis light reaction (GO:0019684), response to oxidative stress (GO:0006979), nucleosome assembly (GO:0006334), lignin biosynthetic process (GO:0009809), generation of precursor metabolites and energy (GO:0006091) and chromatin remodeling (GO:0006338). In contrast, upregulated genes were primarily linked to responses to abscisic acid (GO:0009737), alcohol (GO:0097305), oxygen-containing compounds (GO:1901700), cold acclimation (GO:0009631), response to salt (GO:1902074), response to water (GO:0009415), response to acid chemical (GO:0001101), response to organic substance (GO:0010033), response to lipid (GO:0033993) and response to abiotic stimulus (GO:0009628). Taken together, ABA treatment broadly inhibits metabolic processes and structural organization in the embryo, and may simultaneously enhance its adaptive responses to adverse environmental conditions. Moreover, we found that 44 DEGs were involved in responses to ABA (GO:0009737) (Additional file 6: Data S6). These included genes encoding seven TFs, including three ABA-related TFs (AtABI3, AtAREB3, AtABF3), key components of the ABA signaling pathway, such as four SNF1-RELATED PROTEIN KINASE 2 (SnRK2s), and five PROTEIN PHOSPHATASE 2 C (PP2Cs), ten LEA (LATE EMBRYOGENESIS ABUNDANT) proteins, particularly dehydrins, as well as one ABA transporter.

To further explore the roles of the DEGs, we utilized the KEGG online database (Fig. 3B; Additional file 7: Data S7). Out of the 1,772 upregulated and only 396 (22%) and 938 (30%) downregulated DEGs were assigned to KEGG pathways. Downregulated DEGs were predominantly involved in cyanoamino acid metabolism (ko00460), carbon fixation by Calvin cycle (ko00710), glycolysis/gluconeogenesis (ko00010), motor proteins (ko04814), cysteine and methionine metabolism (ko00270), cutin, suberine, and wax biosynthesis (ko00073), ubiquinone and other terpenoid-quinone biosynthesis (ko00130), photosynthesis-antenna proteins (ko00196), photosynthesis (ko00195), phenylpropanoid biosynthesis (ko00940), MAPK signaling pathway—plant (ko04016), plant-pathogen interaction (ko04626), starch and sucrose metabolism (ko00500), plant hormone signal transduction (ko04075), flavonoid biosynthesis (ko00941), biosynthesis of amino acids (ko01230), and biosynthesis of various plant secondary metabolites (ko00999). The upregulated DEGs were involved in protein processing in the endoplasmic reticulum (ko04141), RNA degradation (ko03018), glycolysis/gluconeogenesis (ko00010), MAPK signaling pathway (ko04016), phenylpropanoid biosynthesis (ko00940), spliceosome (ko03040), mRNA surveillance pathway (ko03015), plant hormone signal transduction (ko04075), starch and sucrose metabolism (ko00500), flavonoid biosynthesis (ko00941), and glycerolipid metabolism (ko00561).

### Phytohormonal control of germinating barley embryos in response to ABA

**Fig. 4:**
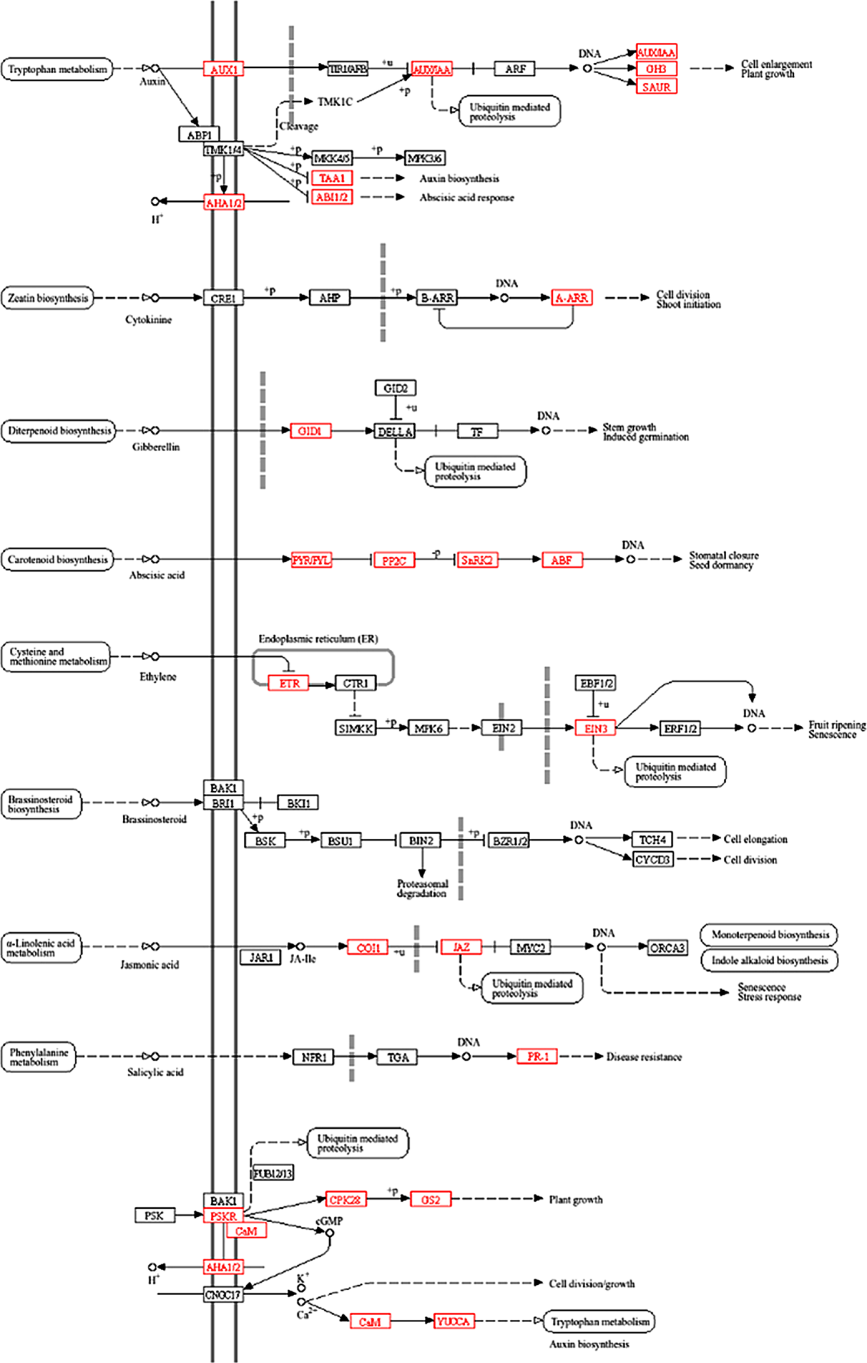
Visualization of plant hormone signal transduction KEGG pathway. Red colour representing upregulation and green colour representing downregulation

Given the well-established role of ABA, with other phytohormones, in regulating seed germination, the ‘plant hormone signal transduction’ pathway has become a key focus of our analysis [1, 50, 51]. The KEGG pathway map illustrates the various plant phytohormone pathways that are differentially regulated in germinating embryos in response to ABA treatment (Fig. 4). Increased activity was observed within the ABA signaling pathway, where elements such as the PYRABACTIN RESISTANCE 1-LIKE (PYR/PYL) receptor family, phosphatases PP2Cs, kinases SnRK2s, and AREB/ABFs transcription factors were identified (Table 2; Additional file 8: Data S8).

**Table 2:** DEGs in germinating barley embryos after ABA treatment linked to plant hormone signal transduction (KEGG)

KEGG identifier	KEGG Name	KEGG Symbol	BaRTv2 ID
K01535	H+-transporting ATPase	AHA1/2 (PMA1/PMA2)	BaRT2v18chr4HG177050
K01915	glutamine synthetase	GS (glnA, GLUL)	BaRT2v18chr2HG105870; BaRT2v18chr4HG178760
K11816	indole-3-pyruvate monooxygenase	YUCCA	BaRT2v18chr5HG232860; BaRT2v18chr2HG108970; BaRT2v18chr3HG147750
K13412	calcium-dependent protein kinase	CPK	BaRT2v18chr5HG269710; BaRT2v18chr3HG153220; BaRT2v18chr4HG177010; BaRT2v18chr5HG238490; BaRT2v18chr5HG269680; BaRT2v18chr5HG256940
K13463	coronatine-insensitive protein 1	COI1	BaRT2v18chr1HG036610
K14432	ABA responsive element binding factor	AREB/ABF	BaRT2v18chr3HG156370; BaRT2v18chr1HG033690
K14488	SAUR family protein	SAUR	BaRT2v18chr7HG378910; BaRT2v18chr7HG374000; BaRT2v18chr6HG319690; BaRT2v18chr6HG292090; BaRT2v18chr2HG105250; BaRT2v18chr2HG105200; BaRT2v18chr2HG105230
K14493	gibberellin receptor GID1	GID1	BaRT2v18chr1HG028980
K14497	protein phosphatase 2 C	PP2C	BaRT2v18chr3HG142490; BaRT2v18chr1HG046520; BaRT2v18chr2HG049520; BaRT2v18chr3HG157400; BaRT2v18chr3HG138810
K14498	serine/threonine-protein kinase SRK2	SnRK2	BaRT2v18chr1HG037480; BaRT2v18chr1HG026070; BaRT2v18chr4HG182300
K14509	ethylene receptor	ETR	BaRT2v18chr6HG314730
K14514	ethylene-insensitive protein 3	EIN3	BaRT2v18chr2HG086440
K02183	calmodulin	CaM	BaRT2v18chr1HG033520; BaRT2v18chr5HG238570
K13449	pathogenesis-related protein 1	PR1	BaRT2v18chr7HG337030; BaRT2v18chr5HG270910; BaRT2v18chr7HG342320; BaRT2v18chr5HG270890; BaRT2v18chr7HG346350; BaRT2v18chr5HG244050
K13464	jasmonate ZIM domain-containing protein	JAZ	BaRT2v18chr2HG106520; BaRT2v18chr2HG082260
K13946	auxin influx carrier (AUX1 LAX family)	AUX1/LAX	BaRT2v18chr4HG185730; BaRT2v18chr5HG271040; BaRT2v18chr5HG226700; BaRT2v18chr1HG013580; BaRT2v18chr5HG264940; BaRT2v18chr5HG226680; BaRT2v18chr3HG148390; BaRT2v18chr3HG124860; BaRT2v18chr7HG366910; BaRT2v18chr7HG339240
K14487	auxin responsive GH3 gene family	GH3	BaRT2v18chr3HG152100; BaRT2v18chr2HG091440; BaRT2v18chr2HG059720
K14492	two-component response regulator ARR-A family	A-ARR	BaRT2v18chr2HG111060; BaRT2v18chr5HG237800; BaRT2v18chr2HG085110; BaRT2v18chr2HG092540
K14496	abscisic acid receptor PYR/PYL family	PYL	BaRT2v18chr1HG034770
K16903	L-tryptophan-pyruvate aminotransferase	TAA1	BaRT2v18chr3HG123080
K27625	phytosulfokine receptor 1	PSKR1	BaRT2v18chr6HG307580

Increased ABA biosynthesis was shown by the upregulation of *NINE-CIS-EPOXYCAROTENOID DIOXYGENASE* (*NCED*) and two genes annotated as *BETA-CAROTENE 3-HYDROXYLASE* (Table 3; Additional file 9: Data S9). Genes from other phytohormonal pathways, including auxin, jasmonic acid (JA), gibberellin (GA), ethylene (ET), cytokinin (CTK), and salicylic acid (SA), were also targeted. In addition, genes involved in calcium signaling pathways, which are crucial for cell division and growth processes, were mapped, along with components of phosphorylation cascades and plasma membrane transport systems that contribute to enhanced growth responses (Table 2; Fig. 4; Additional file 8: Data S8). The KEGG pathway map analysis also showed DEGs within several key pathways, including the biosynthesis of JA, BR, diterpenoids (including the biosynthesis of GA), ET biosynthesis, zeatin, and tryptophan metabolism pathway related to the production of indole-3-acetic acid (IAA) (Table 3; Additional file 9: Data S9). These results suggest a broad network of phytohormonal crosstalk triggered by ABA within the embryo.

**Table 3:** DEGs in germinating barley embryos after ABA treatment linked to plant hormone biosynthesis pathways (KEGG)

KEGG pathway map name	KEGG pathway map ID	KEGG identifier	KEGG Symbol	BaRTv2 ID
Jasmonic acid biosynthesis	map00592	K00454	LOX2S	BaRT2v18chr5HG221560; BaRT2v18chr6HG282910
		K05894	OPR	BaRT2v18chr7HG343660; BaRT2v18chr7HG343690; BaRT2v18chr7HG330350; BaRT2v18chrUnG390320; BaRT2v18chr7HG343680; BaRT2v18chr7HG385100
Brassinosteroid biosynthesis	map00905	K15639	CYP734A1, BAS1	BaRT2v18chr6HG299590; BaRT2v18chr3HG126970; BaRT2v18chr2HG062140
		K20623	CYP92A6	BaRT2v18chr5HG247530; BaRT2v18chr7HG347310
Diterpenoid biosynthesis	map00904	K04122	GA3	BaRT2v18chr7HG368050
		K04125	GA2ox	BaRT2v18chr2HG092430
		K05282	GA20ox	BaRT2v18chr1HG030670
		K16085	CYP99A2_3	BaRT2v18chr2HG052040; BaRT2v18chr2HG055770
Ethylene biosynthesis	map00270	K00789	metK, MAT	BaRT2v18chr6HG310160; BaRT2v18chr6HG310120
		K01762	ACS	BaRT2v18chr3HG124710
		K05933	E1.14.17.4	BaRT2v18chr5HG250670; BaRT2v18chr4HG184710; BaRT2v18chr6HG319390
		K20772	ACS1_2_6	BaRT2v18chr2HG095020
Abscisic acid biosynthesis	map00906	K09840	NCED	BaRT2v18chr5HG223780
		K15746	beta-carotene 3-hydroxylase	BaRT2v18chr2HG094980; BaRT2v18chr4HG215920
Zeatin biosynthesis	map00908	K00279	cytokinin dehydrogenase	BaRT2v18chr1HG019230; BaRT2v18chr5HG246980
		K13495	cis-zeatin O-glucosyltransferase	BaRT2v18chr2HG096460; BaRT2v18chr2HG096430
Tryptophan metabolism	map00380	K01426	E3.5.1.4; amidase	BaRT2v18chr2HG051690; BaRT2v18chr2HG051700
		K11816	YUCCA	BaRT2v18chr5HG232860; BaRT2v18chr2HG108970; BaRT2v18chr3HG147750
		K16903	TAA1	BaRT2v18chr3HG123080
		K22450	SNAT	BaRT2v18chr7HG363550
		K22588	ASMT	BaRT2v18chr1HG001560

### Comparative analysis of transcriptomics and metabolomics in germinating barley embryos in response to ABA

**Fig. 5:**
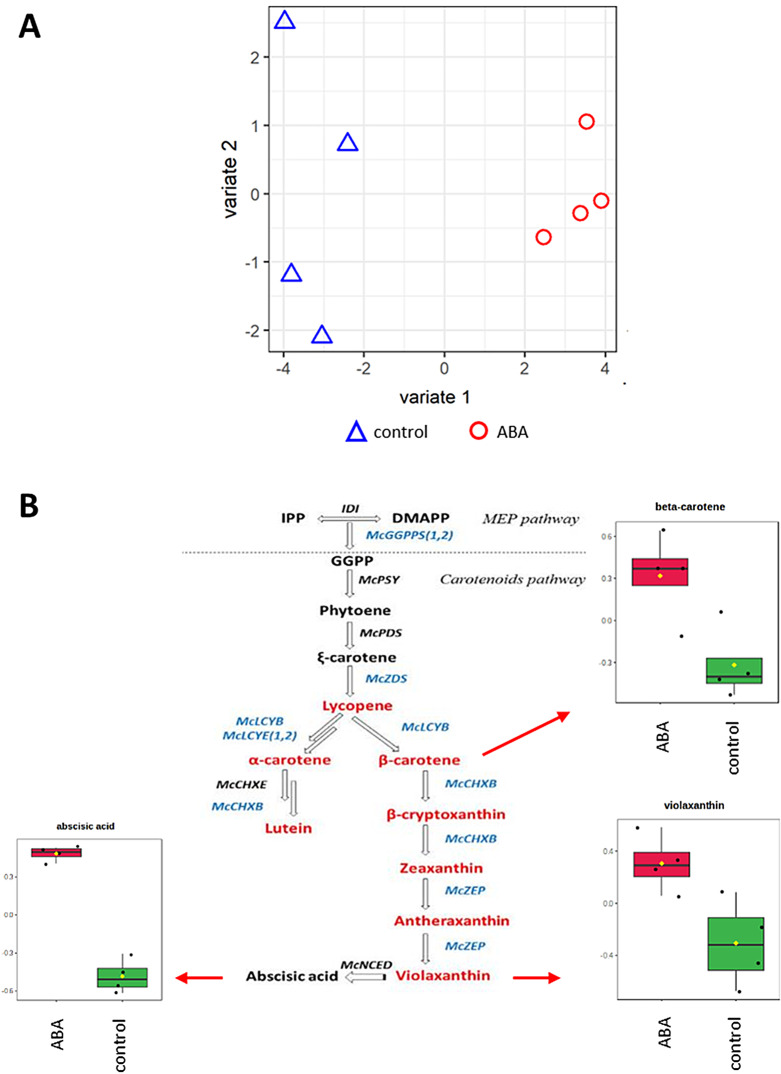
(**A**) Partial Least Squares Discriminant Analysis (PLS-DA) of metabolomic profiles obtained from barley embryos germinating in the presence of 75 µM ABA and in the control conditions. Control samples are represented by blue triangles and ABA samples by red circles. (**B**) Quantitative analysis of key metabolites in the carotenoid biosynthesis pathway in barley embryos germinating with 75 µM ABA compared to the control conditions. Box plots show normalized concentrations of identified metabolites

To complement our transcriptome analysis, we investigated the metabolomic changes in barley embryos under ABA treatment to gain a broader understanding of the molecular response. PLS-DA showed that ABA treatment resulted in a clear metabolic shift in the embryo (Fig. 5A). The top loading vectors for the metabolomic data were related to phytohormonal pathways and 25 showed decreased accumulation, whereas 7 showed increased levels after ABA treatment (Additional file 10: Data S10). Within this phytohormonal groups, ABA pathways were prominent, and it appeared that ABA treatment initiated further endogenous ABA production as shown by statistically significant increases in violaxanthin (p-value = 0.02; FC = 1.76) and β-carotene (p-value = 0.04; FC = 1.90) (Fig. 5B). When relating these to ABA associated DEGs, 20 were upregulated and 15 were downregulated (Table 4).

**Table 4:** Phytohormone-related DEGs in the transcriptomic block in germinating barley embryos after ABA treatment

BaRTv2 ID	Gene annotation	log2FC	KEGG ID	KEGG symbol
BaRT2v18chr6HG307580	Phytosulfokine receptor 1	-2.18	K27625	PSKR1
BaRT2v18chr1HG018060	Esterase/lipase/thioesterase-like protein	1.51	K27538	PES
BaRT2v18chr7HG349770	Diacylglycerol O-acyltransferase 2	1.86	K22848	DGAT2
BaRT2v18chr3HG119330	Lipid phosphate phosphatase-like protein	2.01	K18693	DPP1, DPPL, PLPP4_5
BaRT2v18chr3HG123080	Tryptophan aminotransferase	-1.83	K16903	TAA1
BaRT2v18chr2HG086440	Ethylene-insensitive 3	5.44	K14514	EIN3
BaRT2v18chr6HG314730	Ethylene receptor	3.20	K14509	ETR
BaRT2v18chr1HG037480	SnRK2 serine threonine protein kinase	-2.91	K14498	SnRK2
BaRT2v18chr4HG182300	SnRK2 serine threonine protein kinase	1.76	K14498	SnRK2
BaRT2v18chr3HG138810	Protein-serine/threonine phosphatase	1.91	K14497	PP2C
BaRT2v18chr1HG034770	Abscisic acid receptor	-2.46	K14496	PYL
BaRT2v18chr1HG028980	Gibberellin receptor GID1a	2.05	K14493	GID1
BaRT2v18chr2HG092540	Response regulatory domain-containing protein	-2.80	K14492	A-ARR
BaRT2v18chr2HG105230	Auxin responsive SAUR protein	1.55	K14488	SAUR
BaRT2v18chr6HG292090	Auxin responsive SAUR protein	-3.60	K14488	SAUR
BaRT2v18chr2HG059720	GH3 family protein	-2.45	K14487	GH3
BaRT2v18chr7HG339240	Auxin-responsive protein	-4.03	K14484	AUX/IAA
BaRT2v18chr1HG033690	BZIP transcription factor	1.75	K14432	AREB/ABF
BaRT2v18chr4HG185730	Auxin influx transporter	-3.72	K13946	AUX1/LAX
BaRT2v18chr5HG276890	Putative glycerol-3-phosphate 1-O-acyltransferase	3.59	K13508	GPAT
BaRT2v18chr2HG082260	Tify domain-containing protein	-3.50	K13464	JAZ
BaRT2v18chr1HG036610	Coronatine insensitive protein 1	1.61	K13463	COI1
BaRT2v18chr5HG244050	Pathogenesis-related protein class 1	-8.06	K13449	PR1
BaRT2v18chr6HG299270	Respiratory burst oxidase	1.78	K13447	RBOH
BaRT2v18chr5HG256940	Calcium-dependent protein kinase	2.34	K13412	CPK
BaRT2v18chr5HG269680	Calcium-dependent protein kinase	-9.00	K13412	CPK
BaRT2v18chr2HG108970	Flavin-containing monooxygenase	-4.96	K11816	YUCCA
BaRT2v18chr3HG147750	Flavin-containing monooxygenase	1.80	K11816	YUCCA
BaRT2v18chr7HG368430	O-acyltransferase	2.39	K11155	DGAT1
BaRT2v18chr2HG052310	Heat shock protein	3.42	K04079	HSP90A, htpG
BaRT2v18chr5HG238570	Calmodulin protein	-4.70	K02183	CaM
BaRT2v18chr2HG105870	Glutamine synthetase	-2.14	K01915	GS (glnA, GLUL)
BaRT2v18chr4HG178760	Glutamine synthetase	2.03	K01915	GS (glnA, GLUL)
BaRT2v18chr4HG177050	Plasma membrane ATPase	3.55	K01535	AHA1/2 (PMA1/PMA2)
BaRT2v18chr1HG027420	Diacylglycerol kinase	2.92	K00901	dgkA, DGK

**Fig. 6:**
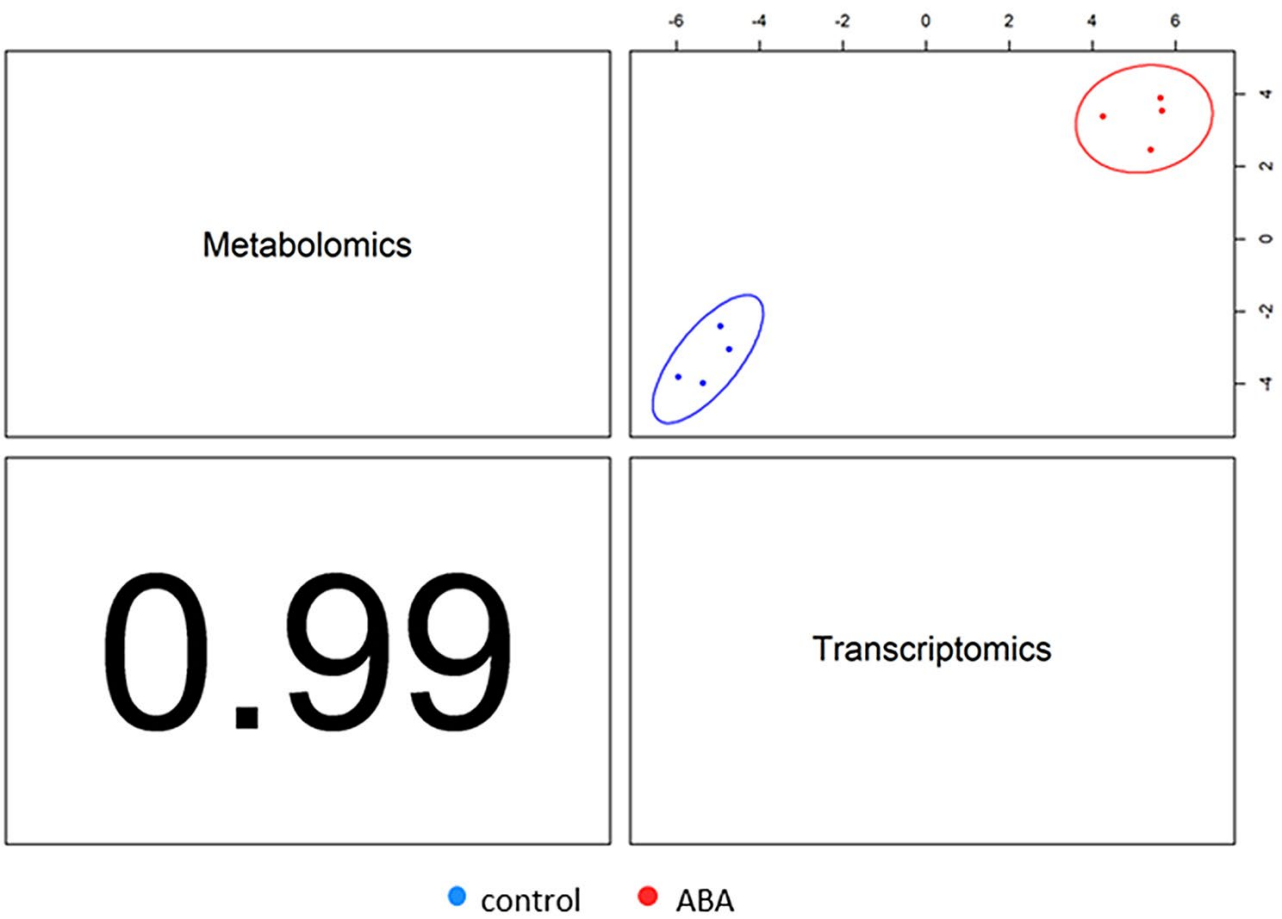
Correlation between metabolomic and transcriptomic data from barley embryos germinating in the presence of 75 µM ABA and in the control conditions. Control samples are shown in blue, and ABA samples in red

**Fig. 7:**
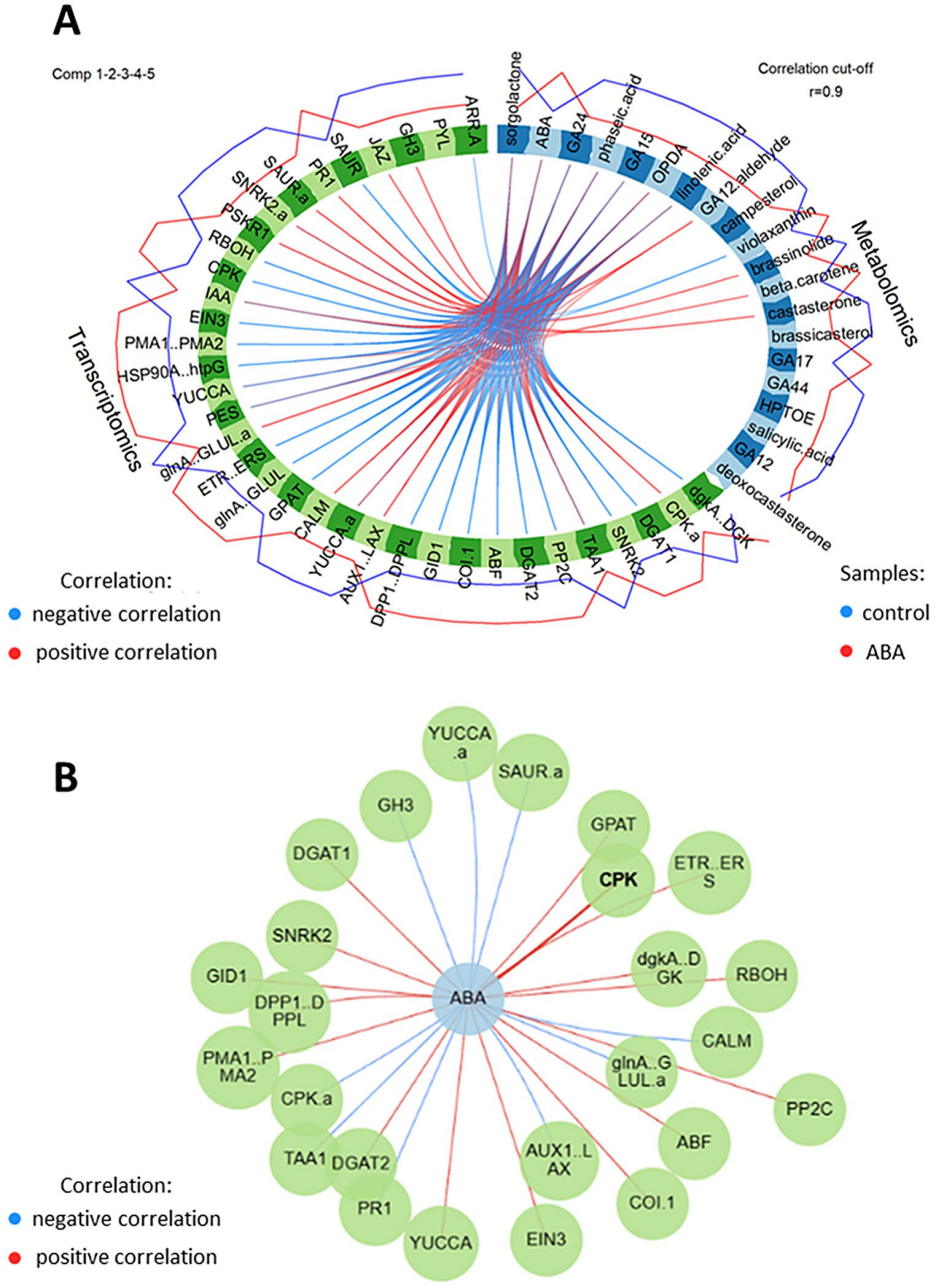
Interactions between hormone networks. (**A**) Circos plot showing correlations between key transcriptomic and metabolomic parameters in samples obtained from barley embryos germinating in the presence of 75 µM ABA and in the control conditions. Red lines within the circos plot indicate positive and blue lines represent negative correlations. The red line outside the circus plot shows levels in ABA-treated samples, and the blue line represents levels in control samples. Correlation cut-off *r* = 0.9. (**B**) Abscisic acid (blue circle) interactions with genes (green circles). *ARR-A* (*BaRT2v18chr2HG092540*), *PYL* (*BaRT2v18chr1HG034770*), *GH3* (*BaRT2v18chr2HG059720*), *JAZ* (*BaRT2v18chr2HG082260*), *SAUR* (*BaRT2v18chr2HG105230*), *PR1* (*BaRT2v18chr5HG244050*), *SAUR.a* (*BaRT2v18chr6HG292090*), *SNRK2.a* (*BaRT2v18chr1HG037480*), *PSKR1* (*BaRT2v18chr6HG307580*), *RBOH* (*BaRT2v18chr6HG299270*), *CPK* (*BaRT2v18chr5HG256940*), *IAA* (*BaRT2v18chr7HG339240*), *EIN3* (*BaRT2v18chr2HG086440*), *PMA1.PMA2* (*BaRT2v18chr4HG177050*), *HSP90A.htpG* (*BaRT2v18chr2HG052310*), *YUCCA* (*BaRT2v18chr3HG147750*), *PES* (*BaRT2v18chr1HG018060*), *glnA.GLUL.a* (*BaRT2v18chr2HG105870*), *ETR.ERS* (*BaRT2v18chr6HG314730*), *glnA.GLUL* (*BaRT2v18chr4HG178760*), *GPAT* (*BaRT2v18chr5HG276890*), *CALM* (*BaRT2v18chr5HG238570*), *YUCCA.a* (*BaRT2v18chr2HG108970*), *AUX1.LAX* (*BaRT2v18chr4HG185730*), *DPP1.DPPL* (*BaRT2v18chr3HG119330*), *GID1* (*BaRT2v18chr1HG028980*), *COI.1* (*BaRT2v18chr1HG036610*), *ABF* (*BaRT2v18chr1HG033690*), *DGAT2* (*BaRT2v18chr7HG349770*), *PP2C* (*BaRT2v18chr3HG138810*), *TAA1* (*BaRT2v18chr3HG123080*), *SNRK2* (*BaRT2v18chr4HG182300*), *DGAT1* (*BaRT2v18chr7HG368430*), *CPK.a* (*BaRT2v18chr5HG269680*), *dgkA.DGK* (*BaRT2v18chr1HG027420*)

The ABA catabolic pathway was also altered, supported by reduced levels of the major catabolic metabolite of ABA-phaseic acid (PA). This is consistent with the increased expression of genes encoding key enzymes in the ABA biosynthesis pathway, *9-CIS-EPOXYCAROTENOID DIOXYGENASE* (*NCED*), and *BETA-CAROTENOID HYDROXYLASES*. In addition, ABA signaling-related genes, such as *PYR/PYL*, *PP2Cs*, *SnRK2s* and *AREB/ABFs* also showed altered expression (Table 2; Additional file 8: Data S8; Table 3; Additional file 9: Data S9). In addition to ABA, other phytohormones also play a significant role in differentiating responses in both metabolomic and transcriptomic data. Metabolites related to gibberellins (GA12, GA12-aldehyde, GA15, GA17, GA24, and GA44), brassinosteroids (brassinolide, castasterone, deoxocastasterone, and campesterol), salicylic acid, jasmonates (OPDA, linolenic acid, and HPTOE), and strigolactones (sorgolactone) were major sources of variation. Considering DEGs, these included transcripts from the GA, SA, JA, CTK, and auxin pathways suggests the involvement of these phytohormones in the regulation of seed germination in response to ABA (Table 4; Additional file 10: Data S10). Integration and comparison of the phytohormone associated DEGs and metabolites suggested a high degree of correlation (0.99) (Fig. 6). The key associations are illustrated using circos plot using a correlation coefficient cutoff of > 0.9 to indicate both positive and negative associations (Fig. 7A; Additional file 11: Data S11). The gene correlations with ABA were highlighted in a network centered on ABA (Fig. 7B). Unsurprisingly, ABA levels were positively correlated with the key signaling components *SnRK2*, *ABF* and *PP2C*. ET (*ETR/ERS*, *EIN3*), jasmonate (*COI1*) and gibberellin (*GID1*) associated gene expression also positively correlated with ABA. Also positively correlated with ABA were genes linked to lipid-associated events (*DGKA*,* DGAT1*,* DPP1*,* DGAT2*,* GPAT*). A negative correlation was seen with most auxin-associated genes (*SAUR.a*,* YUCCA.a*,* TAA1*,* AUX1/LAX*) and also with *PR1*, which is a marker for salicylate effects.

### Insights from bulk RNA-seq and spatial transcriptomics into ABA-dependent genetic regulation of seed development and germination

**Fig. 8:**
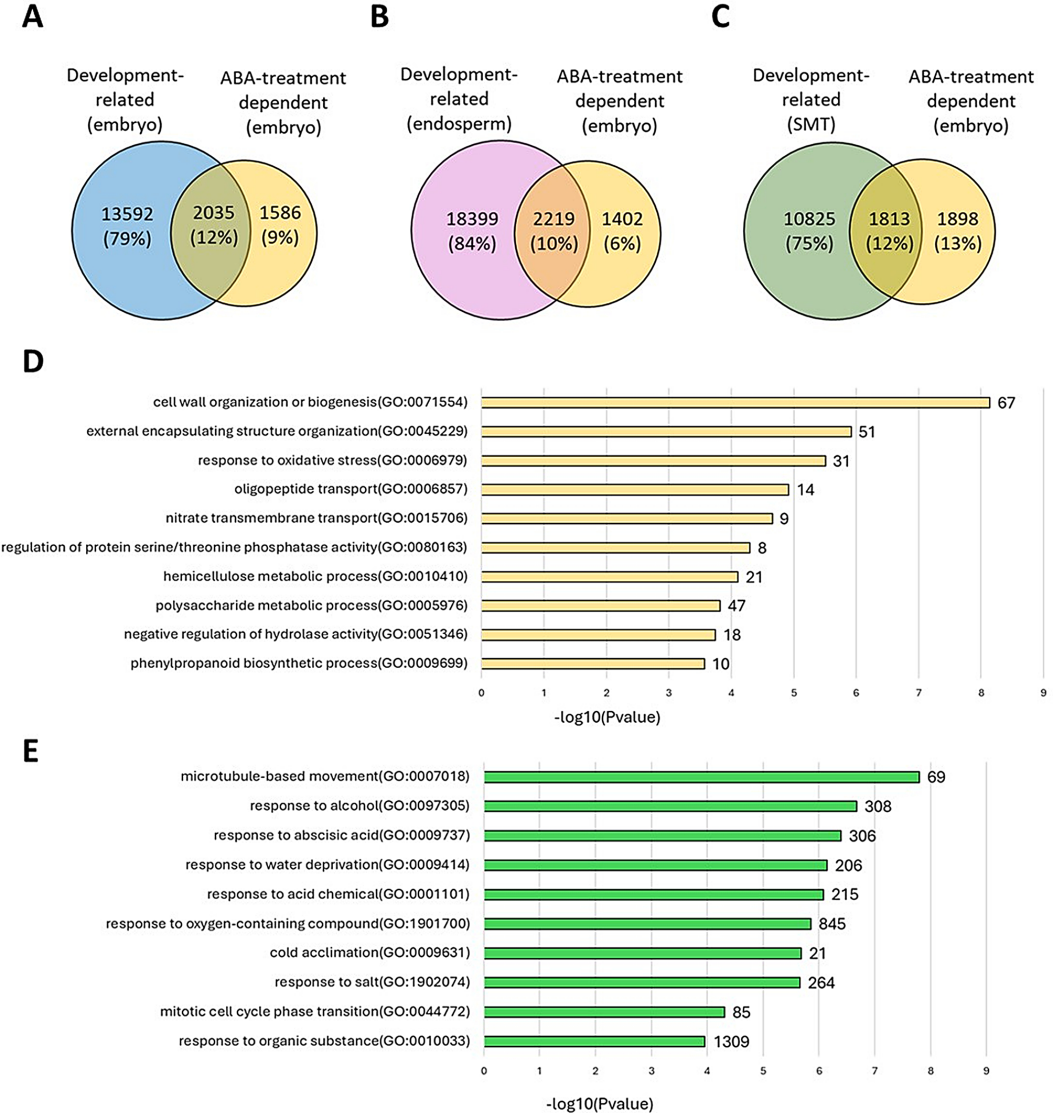
Venn diagram illustrating the common and unique differentially expressed genes (DEGs) in barley embryos germinating under 75 µM ABA treatment, in comparison to DEGs in (**A**) embryo, (**B**) endosperm, (**C**) seed maternal tissue (SMT) during seed development. (**D**) Overrepresented GO biological processes of ABA-treatment dependent DEGs in embryo. (**E**) Overrepresented GO biological processes of ABA-treatment dependent and development-related DEGs in embryo

Next, we compared the transcriptomic profiles of ABA-treated barley embryos with those of developing seeds. For this purpose, we selected 3,621 DEGs with HORVU.MOREX identifiers from our RNA-seq data. This dataset was compared to the DEGs identified by Kovacik et al. (2024), where 15,627 DEGs were detected in the embryo, 20,618 DEGs in the endosperm, and 12,638 DEGs in the seed maternal tissue (SMT) during seed development [44]. Comparative analysis of these datasets revealed common genes between ABA-treated germinating embryos and individually developing seed tissues: 2,035 genes, 2,219 genes, and 1,813 genes in the embryo, endosperm and SMT, respectively. In addition, ABA treatment-dependent DEGs were identified in each of these tissues: 1,586 genes in the embryo, 1,402 genes in the endosperm, and 1,898 genes in the SMT (Fig. 8A, B, C). Next, focusing on the embryo tissue, we analyzed GO-BP functions for 1,586 ABA treatment-dependent DEGs (Additional file 12: Data S12). The most enriched GO biological processes were cell wall organization and cell structure modification, such as cell wall organization or biogenesis (GO:0071554), external encapsulating structure organization (GO:0045229), hemicellulose metabolic process (GO:0010410), polysaccharide metabolic process (GO:0005976). The processes related to the response to stress were also altered: response to oxidative stress (GO:0006979), phenylpropanoid biosynthetic process (GO:0009699), oligopeptide transport and metabolism (GO:0006857), nitrate transmembrane transport (GO:0015706), regulation of enzymatic activity (GO:0080163), and negative regulation of hydrolase activity (GO:0051346) (Fig. 8D; Additional file 13: Data S13; Additional file 14: Data S14). Among the DEGs identified, 2,035 overlapped with the DEGs expressed in the embryo during seed development, as reported by Kovacik et al. (2024) [44] (Additional file 15: Data S15). This overlap highlights the conserved processes regulated by ABA in stress responses, as well as during seed development. GO enrichment analysis revealed that the functions of these genes are linked to cell movement and division (e.g., microtubule-based movement (GO:0007018), mitotic cell cycle phase transition (GO:0044772). Furthermore, GO-BP processes related to stress and abiotic factor responses were enriched, such as response to abscisic acid (GO:0009737), response to water deprivation (GO:0009414), cold acclimation (GO:0009631), and response to salt (GO:1902074), among others (Fig. 8E; Additional file 16: Data S16; Additional file 17: Data S17). The response to ABA (GO:0009737) represents a common and critical process shared between ABA treatment and embryo development.

**Fig. 9:**
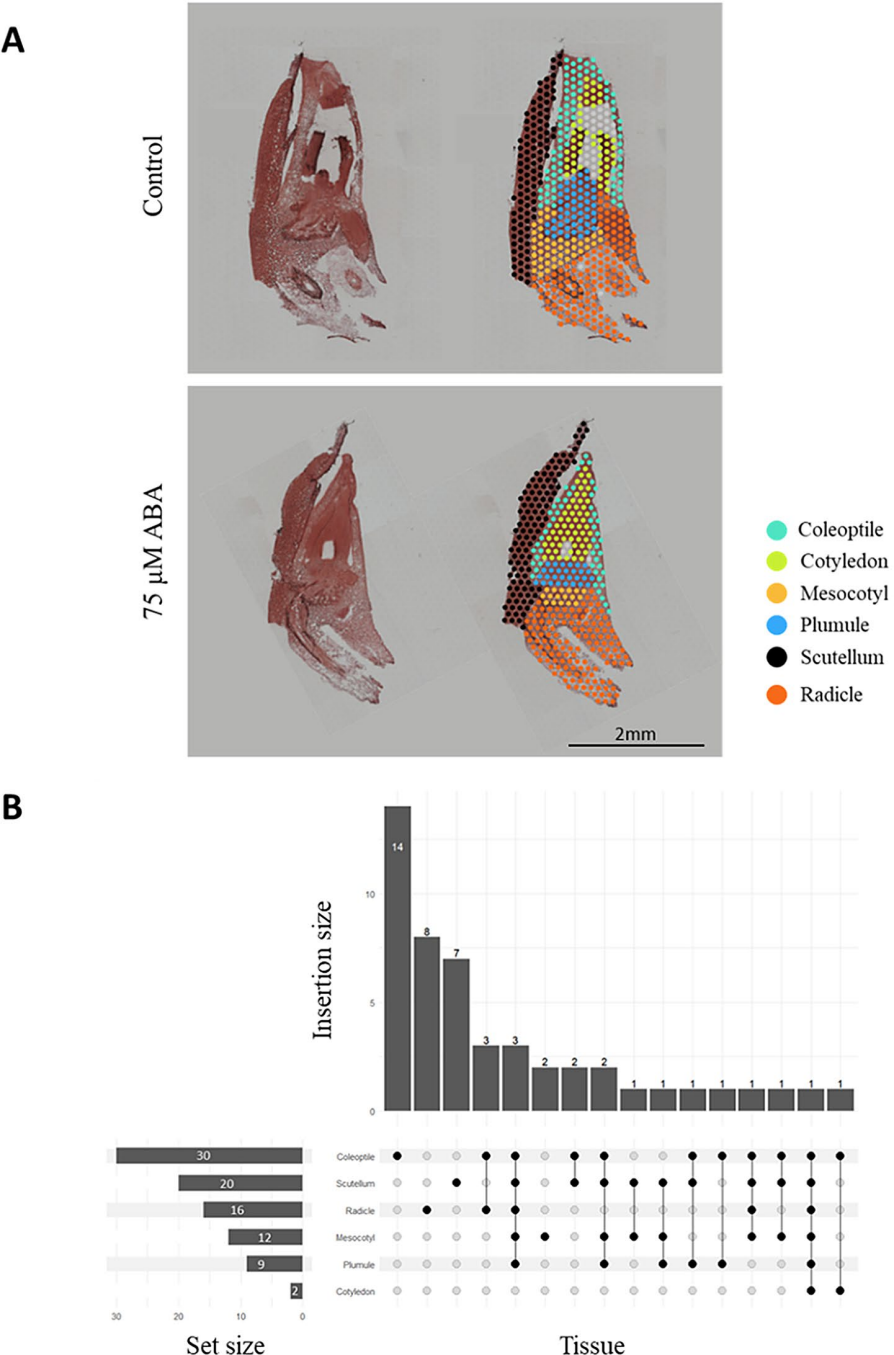
Spatial transcriptomics analysis of germinating embryos under control conditions and in the presence of ABA. (**A**) Histological visualization of embryos and spatial representation of cluster localization. (**B**) Distribution of ABA-treatment dependent differentially expressed genes (DEGs) detected in RNA-seq and spatial transcriptomics experiment across embryo tissues

To extend our analysis beyond bulk RNA-seq and capture spatial gene expression patterns, we performed Visium spatial transcriptomics (10× Genomics) to gain deeper insights into tissue-specific ABA-responsive gene expression. We were able to precisely localize gene expression across six germinating embryo tissues in response to ABA: coleoptile, cotyledon, mesocotyl, plumule, scutellum, and radicle (Fig. 9A). Among 1,586 ABA treatment-dependent DEGs identified in our bulk RNA-seq experiment, we assigned tissue-specific expression to 49 DEGs (Fig. 9B). 30 of these genes were expressed in the coleoptile, 20 in the scutellum, 16 in the radicle, 12 in the mesocotyl, 9 in the plumule, and 2 in the cotyledon. The coleoptile tissue expressed the largest number of tissue-specific genes, accounting for 14 DEGs. Single tissue expression was also observed for 8 DEGs in the radicle, 7 DEGs in the scutellum, and 2 DEGs in the mesocotyl. Additionally, 18 DEGs were present in more than one tissue possibly indicated ABA-dependent genes have common functions in different embryonic regions. A comparison of bulk RNA-seq and spatial transcriptomics results revealed a high agreement in the overall gene expression pattern, indicating a consistency of results obtained using both technologies (Table 5).

**Table 5:** Expression profiles of ABA-dependent DEGs in RNA-seq and Spatial transcriptomics across germinating embryo tissues

BaRTv2 ID	log2FC	Gene annotation	log2FC					
	RNA-seq		Coleoptile	Cotyledon	Mesocotyl	Plumule	Scutellum	Radicle
BaRT2v18chr7HG337790	8.59	GDSL esterase/lipase	6.95	#N/D	#N/D	#N/D	#N/D	#N/D
BaRT2v18chr1HG031670	4.82	Wound-induced protease inhibitor	-2.80	#N/D	#N/D	#N/D	#N/D	#N/D
BaRT2v18chr5HG240300	3.30	Asparagine synthetase	4.54	7.54	3.82	6.94	5.67	3.70
BaRT2v18chr3HG162300	2.99	Selenium-binding protein	1.91	#N/D	2.87	4.24	2.36	3.65
BaRT2v18chr3HG124910	2.42	Clavaminate synthase-like protein	3.66	#N/D	#N/D	#N/D	#N/D	#N/D
BaRT2v18chr7HG342110	2.40	Sucrose synthase	#N/D	#N/D	-1.48	#N/D	#N/D	#N/D
BaRT2v18chr2HG059360	2.33	Hypoxia-responsive family protein-like	1.17	#N/D	0.83	0.77	1.27	#N/D
BaRT2v18chr3HG149970	2.03	Aspartate aminotransferase	#N/D	#N/D	#N/D	#N/D	-0.45	#N/D
BaRT2v18chr7HG365400	1.97	Phosphatidylinositol 4-kinase gamma 4	0.69	#N/D	#N/D	#N/D	#N/D	#N/D
BaRT2v18chr1HG047280	1.77	Glucose-1-phosphate adenylyltransferase	2.97	#N/D	#N/D	3.16	2.36	#N/D
BaRT2v18chr5HG264980	1.75	Sulfate adenylyltransferase	3.74	#N/D	0.89	3.18	2.13	2.65
BaRT2v18chr3HG145450	1.71	Glycosyltransferase	8.50	#N/D	3.78	6.68	4.97	1.39
BaRT2v18chr3HG141170	1.66	Siroheme synthase	3.68	#N/D	1.96	5.43	2.28	#N/D
BaRT2v18chr3HG155900	1.63	Early response to dehydration 15-like protein	1.56	#N/D	#N/D	#N/D	#N/D	#N/D
BaRT2v18chr5HG237100	1.58	Pyruvate kinase	#N/D	#N/D	#N/D	#N/D	-0.41	#N/D
BaRT2v18chr1HG013760	1.55	Actin cross-linking protein	-0.68	#N/D	#N/D	#N/D	#N/D	-1.49
BaRT2v18chr7HG376830	1.55	Glycosyltransferase	5.07	#N/D	#N/D	#N/D	3.79	#N/D
BaRT2v18chr4HG209640	1.51	Evolutionarily conserved C-terminal region 2	1.28	#N/D	#N/D	1.35	#N/D	#N/D
BaRT2v18chr2HG086550	-1.60	Aldose 1-epimerase	-2.16	#N/D	#N/D	#N/D	#N/D	#N/D
BaRT2v18chr4HG179080	-1.62	UDP-glucose 6-dehydrogenase 4	-1.57	#N/D	#N/D	#N/D	#N/D	#N/D
BaRT2v18chr2HG064340	-1.67	Chymotrypsin inhibitor	#N/D	#N/D	#N/D	#N/D	#N/D	1.42
BaRT2v18chr2HG072330	-1.77	Xyloglucan endotransglucosylase/hydrolase	-0.52	#N/D	#N/D	#N/D	#N/D	#N/D
BaRT2v18chr5HG232080	-1.82	Acyl carrier protein	-1.35	-1.73	#N/D	#N/D	#N/D	#N/D
BaRT2v18chr7HG373140 -BaRT2v18chr7HG373150	-2.04	Peptidyl-prolyl cis-trans isomerase	-0.31	#N/D	#N/D	#N/D	#N/D	-0.99
BaRT2v18chr2HG064310	-2.12	Chymotrypsin inhibitor	#N/D	#N/D	#N/D	#N/D	#N/D	2.44
BaRT2v18chr4HG195760	-2.13	RNA-binding protein	-1.31	#N/D	#N/D	#N/D	#N/D	#N/D
BaRT2v18chr7HG365210	-2.14	Chaperone DnaJ	-1.50	#N/D	#N/D	#N/D	#N/D	#N/D
BaRT2v18chr1HG031720	-2.29	Phospholipase A1	-1.02	#N/D	#N/D	#N/D	#N/D	-0.55
BaRT2v18chr2HG058180	-2.29	Stress responsive protein	#N/D	#N/D	#N/D	#N/D	-4.71	#N/D
BaRT2v18chr5HG269020	-2.45	Agglutinin domain-containing protein	0.94	#N/D	#N/D	#N/D	#N/D	#N/D
BaRT2v18chr6HG320150	-2.47	Alpha-amylase	#N/D	#N/D	#N/D	#N/D	-0.81	#N/D
BaRT2v18chr3HG163760	-2.68	Metacaspase-1	-2.76	#N/D	-2.31	#N/D	-1.27	-0.92
BaRT2v18chr7HG374840	-2.72	Xyloglucan endotransglucosylase	#N/D	#N/D	#N/D	#N/D	-2.89	#N/D
BaRT2v18chr3HG170990	-2.91	Abscisic stress ripening protein	#N/D	#N/D	-2.76	#N/D	-2.37	#N/D
BaRT2v18chr1HG045950	-2.92	Subtilisin-like protease	#N/D	#N/D	-3.03	-3.81	-2.96	#N/D
BaRT2v18chr3HG123190	-3.01	Inhibitor protein	#N/D	#N/D	#N/D	#N/D	#N/D	-0.61
BaRT2v18chr4HG220400	-3.04	Metacaspase-1	-2.41	#N/D	-3.16	#N/D	-1.29	#N/D
BaRT2v18chr3HG174080	-3.17	Jasmonate induced protein	#N/D	#N/D	#N/D	#N/D	-4.22	#N/D
BaRT2v18chr1HG016260	-3.85	Ribulose bisphosphate carboxylase small chain	-2.31	#N/D	#N/D	#N/D	-3.62	#N/D
BaRT2v18chr1HG041160	-3.93	Metal ion binding protein	#N/D	#N/D	#N/D	#N/D	-1.09	#N/D
BaRT2v18chr1HG003900	-4.07	Cinnamyl-alcohol dehydrogenase	-2.39	#N/D	#N/D	#N/D	#N/D	#N/D
BaRT2v18chr2HG062560	-4.13	Cold shock protein	#N/D	#N/D	#N/D	#N/D	#N/D	-4.58
BaRT2v18chr1HG007760	-4.35	Annexin	#N/D	#N/D	-5.35	#N/D	#N/D	#N/D
BaRT2v18chr7HG371000	-4.59	Germin-like protein 8 − 4	-1.41	#N/D	#N/D	#N/D	#N/D	#N/D
BaRT2v18chr7HG378630	-4.60	Chitinase	#N/D	#N/D	#N/D	#N/D	#N/D	-4.76
BaRT2v18chr1HG003860	-4.69	Glycosyltransferase	-2.06	#N/D	#N/D	#N/D	#N/D	#N/D
BaRT2v18chr6HG281850	-4.97	Thionin	#N/D	#N/D	#N/D	#N/D	#N/D	-2.16
BaRT2v18chr7HG374060	-5.35	Endoglucanase	#N/D	#N/D	#N/D	#N/D	#N/D	-5.74
BaRT2v18chr1HG001750	-5.73	Thionin-2.2	#N/D	#N/D	#N/D	#N/D	#N/D	-6.27

## Discussion

In our study, ABA treatment was shown to reduce the expression of a significant number of DEGs in germinating barley embryos. A similar effect was observed in *Arabidopsis thaliana* embryos and germinating wheat embryos, where ABA treatment also led to strong repression of the expression of most genes by 62% and 59%, respectively [52, 53]. These results suggest that ABA acts as a key gene-repressive regulator in embryos. The impact of ABA treatment on transcriptional regulation is further reflected by its influence on the expression of specific transcription factor families. The largest number of transcription factors was from the MYB family in germinating embryos treated with ABA. Plant MYB proteins are distinguished by the highly conserved MYB domain responsible for DNA binding and are involved in a broad spectrum of biological processes such as plant development, secondary metabolism, hormonal signal transduction, disease resistance, and tolerance to abiotic stresses [54]. MYB TFs also play a vital role in the regulation of seed germination. For example, MYB70 inhibits germination in an ABA-dependent manner by interacting with ABI5 [55]. MYB96 cooperates with ABI4 to control lipid mobilization in embryos [56]. In addition, MYB94 and MYB330 modulate the germination process by affecting ABA-dependent signaling pathways [57, 58]. These observations highlight the role of MYB TFs in transcriptional regulation of the response of germinating embryos to ABA-related hormonal signals. Moreover, in our study, we identified 23 TFs with binding sites within DEGs, including bZIP transcription factors such as AtABI3, AtAREB3 and AtABF3, which encode key ABA response regulators. These TFs transduce ABA signals by binding to specific ABA-responsive elements (ABREs) in promoter regions. This coordinated interaction fine-tunes seed responses under stress conditions, ensuring the precise regulation of ABA-responsive genes involved in the germination process [59–62]. GO and KEGG analyses revealed that ABA treatment suppressed metabolic and biosynthetic processes, such as photosynthesis and lignin biosynthesis, while simultaneously activating pathways related to stress response, phytohormone signaling, and environmental adaptation. This suggests that ABA redirects the plant’s physiological priorities from growth and energy production to enhance its ability to cope with stressful conditions.

Our integrative transcriptomic and metabolomic approach revealed a strong interplay between ABA treatment and a broader phytohormonal network. A three-fold increase in ABA accumulation in germinating embryos in the presence of this phytohormone was associated with an increase in the number of key compounds in the ABA biosynthetic pathway, such as violaxanthin and beta-carotene, with the increased expression of ABA biosynthesis genes, and a significant decrease in phasic acid, which is the main catabolic metabolite of ABA. This was also associated with changes in the ABA signaling pathway, with increases in the expression of *SnRK2s* kinase genes (*BaRT2v18chr1HG026070*, *BaRT2v18chr4HG182300*), which activate ABF/AREB transcription factors (BaRT2v18chr3HG156370, BaRT2v18chr1HG033690), triggering adaptation to stress conditions. Simultaneously, there was a decrease in the expression of one *SnRK2* kinase (*BaRT2v18chr1HG037480*), which is linked to a modulation of the intensity of ABA signaling. In our study, the expression of the *PYL* gene (*BaRT2v18chr1HG034770*) was reduced, while the expression of *PP2C* phosphatases (*BaRT2v18chr3HG142490*, *BaRT2v18chr1HG046520*, *BaRT2v18chr2HG049520*, *BaRT2v18chr3HG157400*, and *BaRT2v18chr3HG138810*) was increased. It is known that the activation of the core ABA signaling pathway in response to ABA starts with the binding of ABA to PYR/PYL receptors, which blocks the action of clade A PP2C phosphatases and initiates the response to ABA [63–65]. However, the expression of genes encoding *PYR/PYL* receptors may be reduced and the expression of genes encoding *PP2Cs* may be increased to prevent the excessive response to ABA-induced stress [66, 67]. Our results align with such a modulatory feedback mechanism, suggesting a careful tuning of ABA signaling under stress conditions.

Given the known antagonistic roles of ABA and gibberellin (GA) in seed germination, GA metabolism was also assessed. After ABA treatment, the expression of *GA3* (*ent-kaurene oxidase*), which catalyzes the three consecutive steps of GA biosynthesis, converting ent-kaurene to ent-kaurenic acid, was increased. In addition, the expression of *GA20ox* (*GA20-oxidase*) and *GA2ox* (*GA2-oxidase*) genes was upregulated. GA20ox plays a key role in biosynthesis, converting precursors to active forms of GA, whereas GA2ox is involved in catabolism, inactivating GA [68, 69]. Despite the upregulated expression of genes associated with both pathways, the levels of gibberellins GA12, GA12-aldehyde, GA44, GA15, and GA24 were reduced and negatively correlated with ABA. This suggests that GA catabolism may predominate over biosynthesis. Interestingly, the expression of the gibberellin receptor, *GID1*, which is responsible for the perception of the active forms of GA, was positively correlated with ABA. It is possible that the upregulation of *GID1* at elevated ABA levels may act as a compensatory mechanism, preparing the seeds for a rapid response to GA signals after the stress conditions have declined and the ABA levels decrease.

In addition to GA, our results revealed a relationship between ABA and other phytohormones. ABA treatment led to decreased levels of JA, which may be due to the observed decreases in jasmonate precursors such as linolenic acid, 13-HPOTE (13-hydroperoxyoctadecatrienoic acid) and OPDA (12-oxophytodienoic acid). Reduced expression of *LOX* genes encoding lipoxygenases, which convert linolenic acid to 13(S)-hydroperoxyoctadecatrienoic acid (13-HPOTE), suggests a lower activity of these enzymes, resulting in reduced production of 13-HPOTE. This compound is converted into 12-oxo-phytodienoic acid (OPDA), a direct precursor of JA. Thus, due to the decreased level of 13-HPOTE, there was also a decrease in OPD. As a result, even with the increased expression of genes encoding 12-oxophytodienoate reductase (OPR), which is responsible for the further steps of OPDA conversion to JA, the lack of precursor metabolites effectively blocks the entire JA production pathway. This is an intriguing result, as research indicates that JA enhances ABA function and that JA biosynthetic gene expression and JA levels increase in response to ABA [70, 71]. This could also be suggested from the positive correlations between ABA and *COI1*, an F-box protein that promotes the transcriptional repression of JAZ [72]. However, some studies have suggested antagonistic roles of ABA and JA. In wheat grains, MeJA inhibits the expression of an ABA biosynthetic gene (*TaNCED1*), reducing ABA levels and releasing dormancy [24]. In Arabidopsis, JA and its precursor OPDA inhibit seed germination, suggesting different JA functions depending on the species [22]. Further, besides activating JA signaling, COI1 can also inhibit ABA-mediated responses through the interaction and repression of transcriptional activation of ABI3 and ABI5 [25]. This was consistent with the increased expression of *ABI3* (*BaRT2v18chr3HG161790*) in our study. Clearly, the exact role of this interaction needs to be studied further.

In the present study, we observed a negative correlation between ABA and AUX-related genes after ABA treatment at the transcriptomic level. ABA has an inhibitory effect on key auxin genes, limiting both biosynthesis genes (*BaRT2v18chr3HG123080* and *BaRT2v18chr2HG108970*), transport (*BaRT2v18chr4HG185730*), signaling (*BaRT2v18chr7HG339240*), and the early response to this phytohormone (*BaRT2v18chr2HG05972*, *BaRT2v18chr6HG292090*). In contrast, a positive correlation between ABA and two AUX-related genes (*BaRT2v18chr2HG105230* and *BaRT2v18chr3HG147750*) was observed. Furthermore, reduced expression of genes from the tryptophan pathway, an essential precursor of auxin, suggests that ABA inhibits the expression of genes related to AUX production. However, our data do not indicate a statistically significant increase or decrease in the level of AUX metabolites after ABA treatment. This may suggest that the observed changes in gene expression reflect local, tissue-specific changes in AUX signaling or metabolism, rather than global changes in their endogenous levels. Interestingly, recent studies have shown that exogenous AUX can act synergistically with JA, enhancing the effect of ABA and delaying germination by modulating the transcription factors AUXIN RESPONSE FACTOR 10 (ARF10) and ARF16 [26, 27]. However, the effect of AUX on germination is dose-dependent and ​​it can both stimulate and inhibit this process [73–76]. What is more, Belin et al. (2009) showed that low concentrations of ABA (2 µM) induce the expression of the *ProIAA2:GUS* marker, while higher concentrations (30 µM) strongly inhibit it [77]. ABA also limits the expression of genes encoding proteins responsible for auxin transport, both the influx carrier *AUXIN RESISTANT 1* (*AUX1*) and the efflux carriers *PIN-FORMED 3* (*PIN3)* and *PIN7*, even under light conditions that usually stimulate their activity [78]. Although the role of AUX in germination is still not fully understood, the obtained results highlight the complexity of the interaction between ABA and AUX.

Campesterol, the precursor of BR, plays a key role in flux through the subsequent steps of the BR pathway [79]. The reduction of its level in our metabolomics data, as well as of active BR such as brassinolide and castasterone, suggests that ABA has an inhibitory effect on BR biosynthesis at its early stages. BRs are known to promote seed germination; therefore, their deficiency supports the action of ABA as an inhibitor of this process and maintains seed dormancy [80–83]. In addition, the reduced expression of genes annotated as *CYP92A6*, a key BR biosynthesis gene, and *BAS1* (*CYP734A1*), involved in BR inactivation, suggests that ABA regulates BR homeostasis by modulating both their synthesis and inactivation pathways [84, 85].

Our observations indicate that ABA inhibits the conversion of 1-aminocyclopropane-1-carboxylate (ACC) to ET by reducing the activity of ACC oxidase (ACO) and decreasing the accumulation of its transcripts, which is consistent with previous studies [86]. Our data showing reduced expression of genes related to the synthesis and metabolism of ACC and its precursors, such as *S-adenosylmethionine synthase* (*SAM synthase*; *BaRT2v18chr6HG310120*) and *methionine adenosyltransferase* (*BaRT2v18chr6HG310160*), as well as genes encoding key enzymes of ET biosynthesis, *ACC synthase* (*ACS*; *BaRT2v18chr3HG124710*, *BaRT2v18chr2HG095020*), and *ACC oxidase* (*ACO*; *BaRT2v18chr5HG250670*, *BaRT2v18chr4HG184710*, *BaRT2v18chr6HG319390*). We also observed a positive association between ABA treatment and the expression of homologs of the ET-activated transcription factor *EIN3* (*BaRT2v18chr2HG086440*) and the ET receptor *ERS2* (*BaRT2v18chr6HG314730*). This suggests a two-sided effect of ABA; on the one hand, ABA inhibits the expression of genes involved in ET synthesis, limiting its production, whereas the plant signaling apparatus may be prepared to respond to this phytohormone. Although the effect of ABA on ET signaling is poorly understood, other studies have indicated that ET regulates seed germination by reducing ABA levels and attenuating ABA signaling. Mutations that reduce ET sensitivity (*etr1*, *ein2*, *ein6*) increase ABA sensitivity and inhibit germination, whereas mutations that increase ET sensitivity (*ctr1*, *eto1*) reduce ABA action, promoting germination [87–89]. However, mutations in ET signaling pathway genes, such as *EIN3*, *EIN4*, *EIN5*, and *EIN7* do not significantly affect ABA sensitivity [89]. Additionally, genes encoding the ET receptors *ERS1* and *ERS2* do not play a significant role in modulating ABA signaling or in ET-related responses in the context of seed germination [90].

Studies have shown that ABA treatment leads to a significant reduction in the expression of CTK signaling genes *ARABIDOPSIS RESPONSE REGULATOR 6* (*ARR6*), *ARR7* and *ARR15* during seed germination [91]. ABA regulates transcription by activating the transcription factor ABI4, which directly binds to its promoters and inhibits their expression. Therefore, the downregulation of A-ARR genes observed in our study is consistent with the mechanism by which ABA suppresses CTK signaling and promotes the inhibition of seed germination. Moreover, ABA has been shown that ABA can affect CTK biosynthesis by decreasing the expression of biosynthetic genes such as *ISOPENTENYLTRANSFERASE 3* (*IPT3*) and *IPT8* [21]. The reduced expression of genes involved in CTK degradation (*BaRT2v18chr1HG019230*; *AtCKX5*), biosynthetic enzymes (*BaRT2v18chr5HG246980*; *AtCYP735A1*), and glucosylating enzymes (*BaRT2v18chr2HG096460*; *AtUGT72E1*, *BaRT2v18chr2HG096430*; *AtUGT84A3*) observed in our study suggest a modulation of the balance between active and inactive forms of CTK at the transcriptomic level in response to ABA.

Our study also suggests that strigolactones play an important role in controlling ABA-dependent seed germination. We observed that the level of sorgolactone, a specific type of strigolactone, was negatively correlated with ABA, and its concentration significantly decreased in germinating embryos after ABA treatment [92]. It is also worth noting that ABA and salicylic acid (SA) are phytohormones with opposite functions [93]. Similarly, the observed negative correlation between SA levels and *PR1* gene expression (*BaRT2v18chr5HG244050*) supported the hypothesis of an antagonistic interaction between these two phytohormones.

Comparisons of expression patterns of three developing seed tissues (embryo, endosperm, and SMT) described by Kovacik et al. (2024) showed that only a small number (from 10 to 12%) of DEGs overlap with those identified in our study [44]. This limited overlap suggests that ABA can induce a transcriptional response shaped by a common developmental ABA regulatory core, while also including tissue-specific regulation. Analysis of the biological processes specific to germinating embryos treated with ABA revealed that exogenous ABA application regulates adaptive and structural mechanisms in barley embryos, such as cell wall modification, hemicellulose and polysaccharide metabolism, and responses to oxidative stress and phenylpropanoid biosynthesis. This suggests that the role of exogenous ABA is not limited solely to triggering adaptation in response to abiotic stress but also includes key functions in the regulation of normal developmental processes [94–96].

To further refine our understanding of these spatially distinct regulatory processes, we applied Visium spatial transcriptomics (10× Genomics). Unlike bulk transcriptome analysis, which captures averaged gene expression across mixed cell populations, this approach enabled precise mapping of the gene expression in specific embryo tissues, uncovering spatial patterns critical for understanding localized responses to ABA. We localized the expression of 49 genes, selected from the pool of 1,586 ABA-treatment-dependent DEGs identified in bulk RNA-seq, across six embryonic tissues, such as coleoptile, cotyledon, mesocotyl, plumule, scutellum, and radicle. Our results highlighted the important role of the coleoptile tissue, which exhibited the greatest overlap of DEGs with other embryo tissues and expressed the highest number of tissue-specific genes (14 DEGs). This suggests its dual function as both a hub for shared stress-responsive genes and a site of unique gene expression patterns. The obtained results show that ABA responses are closely related to tissue localization, which may reflect the different physiological roles of individual tissues for embryo development. It is noteworthy that spatial transcriptomics revealed differential gene expression distributions that would otherwise remain masked in bulk RNA-seq data, offering a refined view of tissue-specific ABA responses and their functional implications during germination. This level of resolution underscores the importance of spatial context in interpreting the functional roles of ABA-responsive genes.

## Conclusions

Using bulk transcriptomics, metabolomics and Visium spatial transcriptomics, we provide first spatially resolved, multi-omic map of barley seed germination under exogenous ABA. The data indicate that ABA limits germination by coordinating its own signalling–metabolite module and by interacting with GA, JA, BR, SA and auxin pathways. Spatial mapping additionally points to the coleoptile as a principal site of ABA-responsive transcription, a pattern not visible in bulk datasets. The resulting list of tissue-specific genes and metabolites associated with growth restraint and stress adaptation provides a useful reference for future physiological and breeding studies.

## Electronic supplementary material

Below is the link to the electronic supplementary material.

Supplementary Material 1: **Additional file 1: Data S1.** List of differentially expressed genes (DEGs) in germinating barley embryos at 1 DAI after 75 µM ABA treatment compared to control conditions. (XLSX 1 302 KB)

Supplementary Material 2: **Additional file 2: Data S2.** List of the differentially expressed transcription factors in germinating barley embryos at 1 DAI after 75 µM ABA treatment compared to control conditions. (XLSX 28 KB)

Supplementary Material 3: **Additional file 3: Data S3.** Differentially expressed transcription factors, with binding sites among the differentially expressed genes (DEGs) in germinating barley embryos at 1 DAI after 75 µM ABA treatment compared to control conditions. (XLSX 3 982 KB)

Supplementary Material 4: **Additional file 4: Data S4.** GO biological process analysis of differentially upregulated /downregulated genes (DEGs) in germinating barley embryos at 1 DAI after 75 µM ABA treatment compared to control conditions. (XLSX 77 KB)

Supplementary Material 5: **Additional file 5: Data S5.** List of genes in top overrepresented GO biological process of differentially upregulated / downregulated genes in germinating barley embryos at 1 DAI after 75 µM ABA treatment compared to control conditions. (XLSX 80 KB)

Supplementary Material 6: **Additional file 6: Data S6.** Differentially expressed genes (DEGs) in germinating barley embryos at 1 DAI after 75 µM ABA treatment compared to control conditions within the response to abscisic acid (GO:0009737) Gene Ontology Biological Process (GO-BP). (XLSX 14 KB)

Supplementary Material 7: **Additional file 7: Data S7.** Functional enrichment of differentially expressed transcription factors in germinating barley embryos at 1 DAI after 75 µM ABA treatment compared to control conditions based on the KEGG pathway category. (XLSX 14 KB)

Supplementary Material 8: **Additional file 8: Data S8.** List of upregulated and downregulated differentially expressed genes (DEGs) in germinating barley embryos at 1 DAI after 75 µM ABA treatment compared to control conditions within the Plant Hormone Signal Transduction map (KEGG). (XLSX 15 KB)

Supplementary Material 9: **Additional file 9: Data S9.** List of upregulated and downregulated differentially expressed genes (DEGs) in germinating barley embryos at 1 DAI after 75 µM ABA treatment compared to control conditions within the biosynthesis of plant hormones pathway maps. (XLSX 14 KB)

Supplementary Material 10: **Additional file 10: Data S10.** Identified phytohormones and metabolites in germinating barley embryos at 1 DAI under 75 µM ABA treatment and control conditions. (XLSX 17 KB)

Supplementary Material 11: **Additional file 11: Data S11.** P-value correlations between metabolomic and transcriptomic data of germinating barley embryos at 1 DAI after 75 µM ABA treatment and in control conditions. (XLSX 37 KB)

Supplementary Material 12: **Additional file 12: Data S12.** List of ABA-treatment dependent differentially expressed genes (DEG) in germinating barley embryos at 1 DAI after 75 µM ABA treatment compared to control conditions. (XLSX 410 KB)

Supplementary Material 13: **Additional file 13: Data S13.** GO biological process analysis of ABA-treatment dependent differentially expressed genes (DEGs) in germinating barley embryos at 1 DAI after 75 µM ABA treatment compared to control conditions. (XLSX 24 KB)

Supplementary Material 14: **Additional file 14: Data S14.** Differentially expressed genes (DEGs) in germinating barley embryos at 1 DAI after 75 µM ABA treatment compared to control conditions in ABA-treatment dependent overrepresented GO biological processes. (XLSX 29 KB)

Supplementary Material 15: **Additional file 15: Data S15.** List of ABA-treatment dependent and development-related differentially expressed genes (DEG) in germinating barley embryos at 1 DAI after 75 µM ABA treatment compared to control conditions and in embryos during seed development. (XLSX 651 KB)

Supplementary Material 16: **Additional file 16: Data S16.** GO biological process analysis of ABA-treatment dependent and development-related differentially expressed genes (DEGs) in germinating barley embryos at 1 DAI after 75 µM ABA treatment compared to control conditions. (XLSX 38 KB)

Supplementary Material 17: **Additional file 17: Data S17.** Differentially expressed genes (DEGs) in germinating barley embryos at 1 DAI after 75 µM ABA treatment compared to control conditions in ABA-treatment dependent and development-related overrepresented GO biological processes. (XLSX 43 KB)

## Data Availability

Data generated or analyzed during this study are included in this published article (and its additional files). The RNA-seq data used in the present study were deposited into EMBL-EBI (EMBL’s European Bioinformatics Institute) in the Array Express repository (https://www.ebi.ac.uk/) under the accession number E-MTAB-13989. The spatial transcriptomic data used in the present study have been deposited into EMBL-EBI (EMBL’s European Bioinformatics Institute) in the Array Express repository (https://www.ebi.ac.uk/) under accession number E-MTAB-14835. Transcriptome data from developing seed tissues, used in the comparative analysis, were obtained from the supplementary materials of the study by Kovacik et al. (2024) [44]. The RNA-seq data generated in the study by Kovacik et al. (2024) are available in the Gene Expression Omnibus (GEO) at https://www.ncbi.nlm.nih.gov/geo/ under accession number GSE233316.

## Abbreviations

ABA: Abscisic acid
AUX: Auxin
BR: Brassinosteroid
CTK: Cytokinin
DAI: Day after imbibition
DEGs: Differentially expressed genes
ET: Ethylene
GA: Gibberellic acid
GO: Gene ontology
JA: Jasmonic acid
KEGG: Kyoto Encyclopedia of Genes and Genomes
SA: Salicylic acid
TF: Transcription factor
TPM: Transcript per million
